# Parameters for the RM1 Quantum Chemical Calculation of Complexes of the Trications of Thulium, Ytterbium and Lutetium

**DOI:** 10.1371/journal.pone.0154500

**Published:** 2016-05-25

**Authors:** Manoel A. M. Filho, José Diogo L. Dutra, Gerd B. Rocha, Alfredo M. Simas, Ricardo O. Freire

**Affiliations:** 1 Pople Computational Chemistry Laboratory, Departamento de Química, Universidade Federal de Sergipe, São Cristóvão, SE, Brazil; 2 Departamento de Química Fundamental, Universidade Federal de Pernambuco, Recife, PE, Brazil; 3 Departamento de Química, CCEN, Universidade Federal da Paraíba, João Pessoa, PB, Brazil; University of Edinburgh, UNITED KINGDOM

## Abstract

The RM1 quantum chemical model for the calculation of complexes of Tm(III), Yb(III) and Lu(III) is advanced. Subsequently, we tested the models by fully optimizing the geometries of 126 complexes. We then compared the optimized structures with known crystallographic ones from the Cambridge Structural Database. Results indicate that, for thulium complexes, the accuracy in terms of the distances between the lanthanide ion and its directly coordinated atoms is about 2%. Corresponding results for ytterbium and lutetium are both 3%, levels of accuracy useful for the design of lanthanide complexes, targeting their countless applications.

## Introduction

The computational chemistry of lanthanide complexes at the semiempirical level started in 1994 with the introduction of the Sparkle model[1, 2], which allowed, for the first time, fast quantum chemical geometry optimizations of relatively large complexes. The Sparkle model filled the important gap of lanthanide complexes modelling[3–9] by opening the possibility of having the complexes’ UV-Vis spectra [10] and ligand field parameters[11, 12] predicted.

In 2004, the model was improved with the introduction of Gaussian functions in its core-core repulsion [13] to make it consistent with the AM1 semiempirical model [14]. This improved the Sparkle model quite substantially, and, later, in 2005, the Sparkle model was fully parameterized within AM1 for thulium [15], ytterbium [16], and lutetium [17]. Since different semiempirical models have different characteristics and scopes of applications, it soon became clear that there would be value in parameterizing the Sparkle model for all of the most used and widely distributed semiempirical models available. Thus, the Sparkle model was parameterized for PM3 [18–20]; for PM6 [21], the first semiempirical model parameterized for almost all stable atoms of the periodic table; for PM7 [22], with an emphasis on materials and solid state calculations; and for our RM1 [23].

Overall, results indicate that all Sparkle models are very accurate only when all the directly coordinated atoms to the lanthanides in the complex are either an oxygen or a nitrogen–usually, the most common bonding situation. However, there are many instances in which other atoms coordinate directly with the lanthanide trications, such as carbon, sulfur, chlorine, bromine, and iodine. Thulium, for example, makes a number of complexes directly coordinated to carbon atoms, such as thulium alkylidene complexes [24], which contain a thulium carbon double bond character with the participation of a π-overlap between the carbon and the thulium center for the stabilization of the complex. Likewise, there was recently a study of the interaction of a complex with DNA, which had three chlorine atoms directly coordinated to an ytterbium atom[25]. Another example is a bis(alkyl) complex of lutetium attached to suitable ligands, which, upon activation by an organoborate, initiates the living polymerization of isoprene with high activity [26]. Furthermore, very different bonding situations may also arise, as in the synthesis of a mononuclear lutetium imido complex, which involves intermediate complexes with chlorine atoms and a cyclooctatetraenyl ring, all directly coordinated to lutetium [27]. These are examples of bonding situations, which the Sparkle models cannot properly address. All that is within the realm of the diversity of recent advances in the applications of complexes of thulium as near infrared emitters [28–30], as contrast agents for magnetic resonance imaging [31], and as catalyzers [32]. Also within the realm of recent advances is the utilization of complexes of ytterbium, again as near infrared emitters [33–35], as single-molecule magnets [36, 37], as catalysts [38–40], as selective biomarkers for cancer cell imaging [41], and as DNA binders [25]. Finally, within the scope of the recent advances is the use of complexes of lutetium as gas sensors [42], as catalysts [43, 44], in organic films with switchable electronic and/or interface properties with external electric field [45]; and as near infrared absorbing electrochromes [46]. These complexes, however, tend to have poor thermal stability, poor photostability, and low mechanical strength, usually requiring that they be incorporated, for example, either into sol-gel [30], or into ordered mesoporous materials via a covalently bonded group[28, 29]. Consequently, there is a strong need for theoretical modelling methods capable of addressing these challenges. Recently, we introduced a major upgrade to the Sparkle model, in order to arrive at a model capable of describing any type of bonds between a lanthanide metal and a ligand, within the framework of our semiempirical model RM1 (Recife Model 1) [47], we called the “RM1 model for the lanthanides” [48, 49].

## Method

### RM1 model for the lanthanides

RM1 is a semiempirical molecular orbital model, with the same algebraic structure of AM1 [14], but reparameterized in 2006 with modern numerical techniques [47]. RM1 is accurate and robust for the types of atoms for which it was originally parameterized: H, C, O, N, P, S, F, Cl, Br, and I. Although this set of atoms looks small, these atoms comprise the vast majority of all atoms present in metal ligands and biomolecules. RM1 was therefore our model of choice for parameterizing the lanthanide trications. In the RM1 model for the lanthanides, we then regard the semiempirical lanthanide atom as an amalgamation of two separate entities: the core and the valence shell. The semiempirical core represents the [Xe]4fn electrons, with n = 12 for Tm, n = 13 for Yb, and n = 14 for Lu. The valence shell is described by three sets of atomic orbitals: 5d, 6s, and 6p, and contains 3 valence electrons. Note that this arrangement is capable of describing only the trications of the lanthanides. Therefore, the present parameterization will be only for complexes of trivalent thulium, ytterbium and lutetium. The RM1 parameters are presented in Table 1.

**Table 1:** Parameters for the RM1 model for the trications of Tm, Yb and Lu.

RM1			
Parameters*	Tm^3+^	Yb^3+^	Lu^3+^
*U*_*ss*_	-21.89086990	-21.98345481	-22.03273901
*U*_*pp*_	-7.25280726	-7.65281483	-7.54227122
*U*_*dd*_	-18.18388472	-18.07189890	-18.22211913
*ξ*_*s*_	1.36914712	1.23980761	1.42530151
*ξ*_*p*_	1.67436524	1.84914445	1.79035291
*ξ*_*d*_	1.71439392	1.48537800	1.64260309
*β*_*s*_	-5.48059297	-5.53294866	-5.52743162
*β*_*p*_	0.07885989	-0.08691310	-0.24486752
*β*_*d*_	-4.32179079	-4.14307914	-4.21439954
*F0SD*	8.32612982	8.36931024	8.17149635
*G2SD*	1.47443231	1.26163240	1.08696746
*POC*	2.76348172	2.60857605	2.20966049
*α*	1.26643084	1.30633487	1.43449824
*ZSN*	1.23838679	1.56809440	1.47598765
*ZPN*	1.82123460	1.85481720	2.13648311
*ZDN*	0.95620514	0.74940206	0.65999999
*a*_*11*_	1.34525718	1.31949238	0.77121217
*b*_*21*_	7.85061445	7.58756216	7.66485117
*c*_*31*_	1.25695098	1.51760480	1.72692475
*a*_*12*_	0.01273251	0.02584779	0.01134961
*b*_*22*_	7.56475264	7.88270120	7.87082149
*c*_*32*_	2.88561368	3.23295336	3.46540150

*Parameters are *s*, *p*, and *d* atomic orbital one-electron one-center integrals U_ss_, U_pp_ and U_dd_; the *s*, *p*, and *d* Slater atomic orbital exponents ***ξ***_*s*_, ***ξ***_*p*_, and ***ξ***_*d*_; the *s*, *p*, and *d* atomic orbital one-electron two-center resonance integral terms *β*_*s*_, *β*_*p*_, and *β*_*d*_; the core-core repulsion term *α*; the two-electron integrals F^0^_SD_, G^2^_Sd_; and the additive term ρ_core_ needed to evaluate core-electron and core-core nuclear interactions; the second set of exponents to compute the one-center integrals ***ξ***_*s*_*’*, ***ξ***_*p*_’, and ***ξ***_*d*_*’*; and the six parameters for the two Gaussian functions.

## Results and Discussion

### Parameterization

In 2006, when we introduced the Sparkle/PM3 model for thulium [18], we further perfected our parameterization procedure to make sure the model would acquire a more robust attribute. So, following this line, we first collected all complexes of Tm(III), Yb(III), and Lu(III) of high crystallographic quality (R<5%) extant in the 2015 release of the Cambridge Crystallographic Database, CSD[50, 51]. Of course, it would be unfeasible to parameterize the method using all ligands found. Therefore, we used a sampling technique in order to pick, from the universe of complexes, two smaller sets to become the parameterization sets. In order to do that, for each of the lanthanide trications, we first associated, to each of its complexes, a number corresponding to a measure of the difficulty of predicting its geometry in order to guarantee that the sets would be balanced between complexes with easy to predict geometries and complexes with those geometries that are more difficult to predict. We chose this number to be a measure of the distance between the crystallographic geometry obtained from the CSD2015 and a fully optimized Sparkle/AM1 geometry. This number, R_i_, is defined for each complex i, in Eq 1, below, as:

$$R_{i}=\sum_{j}\sum_{k}\frac{1}{\sigma_{j}^{dist}}\left|d_{i,j,k}^{CSD}-d_{i,j,k}^{Calc}\right|+\sum_{l}\frac{1}{\sigma^{angle}}\left|\theta_{i,\text{l}}^{CSD}-\theta_{i,\text{l}}^{Calc}\right|$$ (1)

where j is an index that runs over all different types of bonds, for example, Ln-O, Ln-N, Ln-C, etc; k runs over all bonds of type j; *σ*_*j*_^*dist*^ is the standard deviation of all bonds of type j from the universe of complexes; CSD refers to geometric variables, either distances d, or angles θ, obtained from the Cambridge Crystallographic Database; and Calc refers to geometric variables obtained from Sparkle/AM1 calculations; l runs over all angles in complex i and *σ*^*angle*^ is the standard deviation of all angles from all complexes in the universe of complexes. For the angles, there was no need to separate them into types, because they all form a homogeneous set. Subsequently, we carried out a divisive hierarchical clustering technique DIANA [52] on the complexes and obtained the stratified sampling in the form of a dendogram. We then applied an optimum allocation to it and arrived to two sub-sets from the universe set of complexes: a smaller set, which we called the small set and a larger one, we called the large set. We did that for each of the three lanthanides considered: thulium, ytterbium and lutetium. For thulium, the universe of complexes contains 19 complexes, the small set contains 5 complexes and the large set contains 10 complexes. The respective numbers for ytterbium are 60, 13 and 31; and, for lutetium, 47, 6, and 14. The universe, small and large sets for each of the lanthanides are described in Tables A-C in S1 File.

By using a combination of non-linear numerical optimization techniques for each of the lanthanides, we then first minimized the sum of all R_i_ calculated for all complexes of the small set–with the only difference that Calc in Eq 1 now refers to the fully optimized geometry of the intermediary model being considered in the optimization step. After this optimization converged, we then proceeded by minimizing the sum of all R_i_ calculated for all complexes of the large set. We carried out this second minimization of the larger set in order to improve the accuracy of the model. After this second minimization converged, we then considered the RM1 model for each of the lanthanides as terminated. We then proceeded to compute two accuracy measures for the model for each complex i, UME_i_, based on the unsigned mean error defined as:

$$UME_{i}=\frac{1}{n}\sum_{j=1}^{n}\left|d_{i,j}^{CSD}-d_{i,j}^{RM1}\right|$$ (2)

where n is the number of j bonds present in complex i, d are bond distances, CSD is the crystallographic bond distance from CSD and RM1 is the distance for the fully optimized geometry of the complex for the RM1 model for the lanthanide being taken into consideration. The first UME took into consideration only distances between the lanthanide ion and the directly coordinating atoms, and are averaged up for all complexes of the universe of complexes and are called UME_(Ln-L)_s. These lanthanide–directly coordinated ligand atom distances are the most important ones for the calculation of ligand field parameters. The second set of distances, includes all of these plus all distances in the coordination polyhedron, i.e. distances we call L-L’, where L and L’ are any two directly coordinated atoms, averaged out for all complexes of the universe of complexes, and that are called here simply UMEs. All unsigned mean errors are defined mathematically in the interval from zero to infinity. Therefore, in principle, they should follow a gamma distribution function, something that can be verified by means of the one-sample nonparametric Kolmogorov-Smirnoff test, whose p-value must be above 0.05 for the fit of the corresponding UME data to be acceptable within a 95% confidence interval. If the fit passes the test, the mean is statistically justified as an accuracy measure of the model.

**Fig 1:**
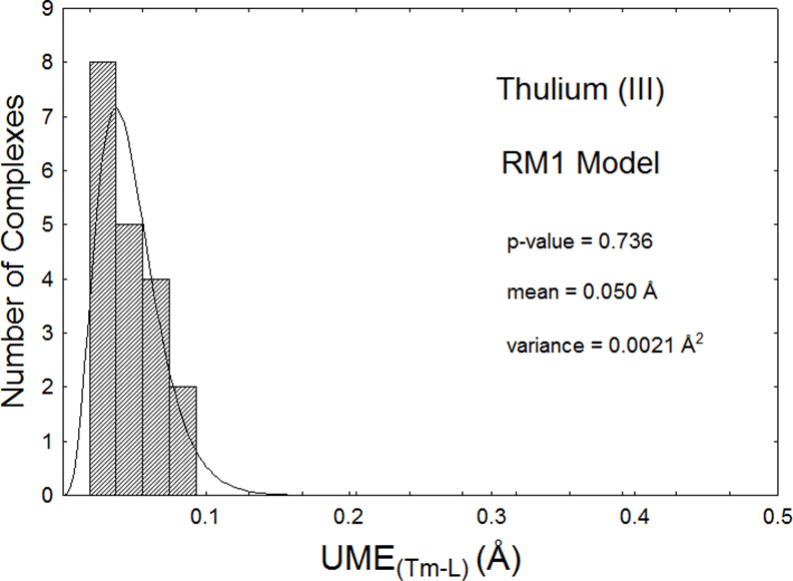
Histogram of the UME_(Tm-L)_s for all 19 complexes of Tm(III) optimized via the RM1 model being advanced in this article. The mean and variance are obtained from the fitted gamma distribution, and the p-value of the one-sample nonparametric Kolmogorov-Smirnoff is also shown. This value is above 0.05, and therefore the data can be considered adjusted to the fitted gamma distribution within a 95% confidence interval.

**Fig 2:**
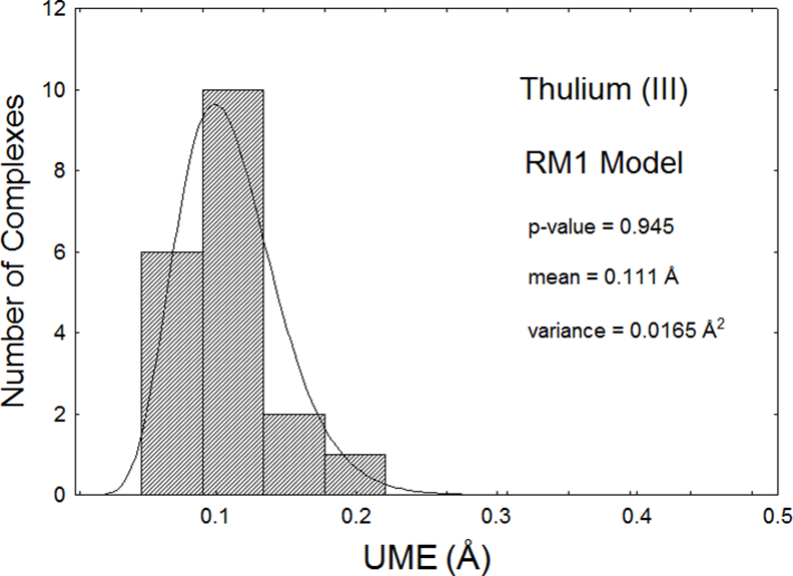
Histogram of the UMEs for all 19 complexes of Tm(III) optimized via the RM1 model being advanced in this article. The mean and variance are obtained from the fitted gamma distribution. The p-value of the one-sample nonparametric Kolmogorov-Smirnoff is also shown. This value is above 0.05, and therefore the data can be considered adjusted to the fitted gamma distribution within a 95% confidence interval.

As an example, Figs 1 and 2 show histograms of UME_(Tm-L)_s and UMEs for the thulium model, superimposed to the corresponding fitted gamma distributions. The p-values are, respectively, 0.736 and 0.945 indicating that the mean UME_(Tm-L)_ of 0.050Å, and mean UME of 0.111Å are good accuracy measures of the RM1 model for thulium. Since the thulium-directly coordinated atom distances mostly lie in the 2.3Å to 2.6Å, this implies that the model is accurate to within 2% for these distances. Corresponding mean UME_(Yb-L)_ and UME_(Lu-L)_ data for the RM1 model for Yb(III) and Lu(III), both equal to 0.08Å, imply that these models are accurate to within 3% for these lanthanides.

### Comparison with the previous Sparkle models

As mentioned in the introduction, the RM1 model for Tm(III), Yb(III), and Lu(III) complexes, is being presented in this article to expand the applicability of the quantum chemical semiempirical modelling of lanthanides to complexes with directly coordinated atoms other than oxygen or nitrogen.

**Fig 3:**
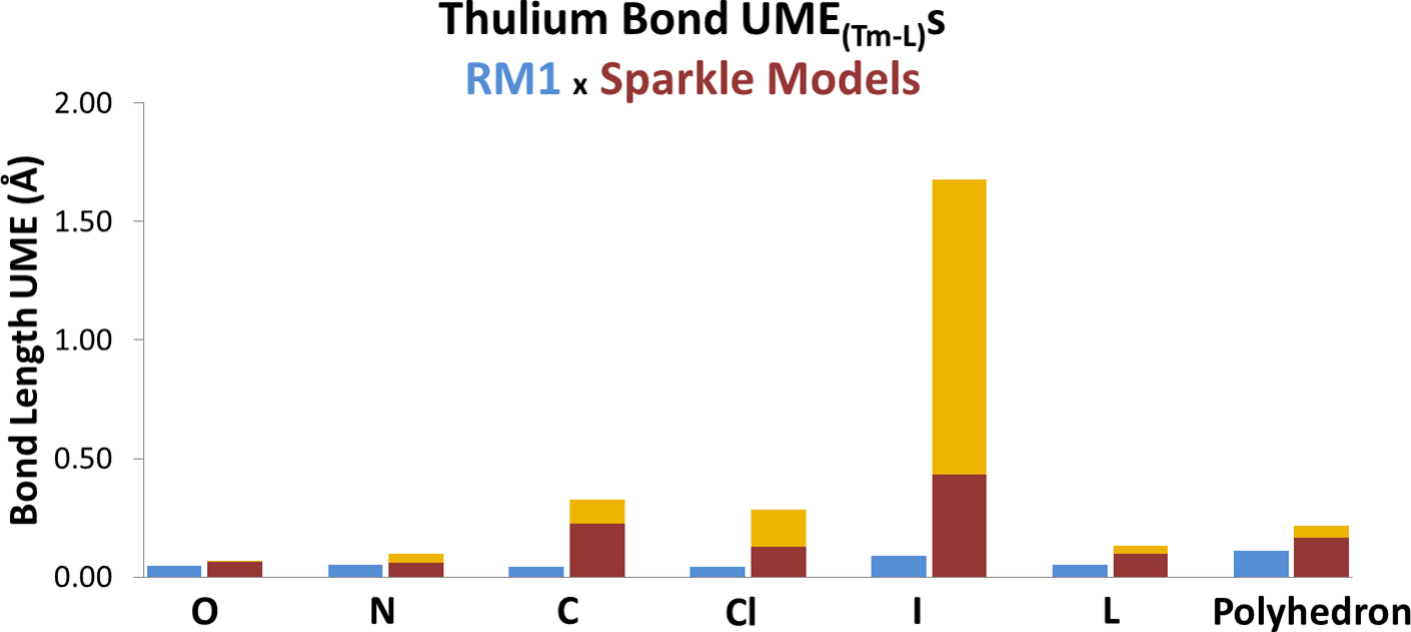
Comparison between the accuracy measures, UME_(Tm-L)_s, of the RM1 model for Tm(III), being advanced in this article (blue bars) and the various Sparkle models (two-lightness bars) for the various types of directly coordinated bonds, indicated on the horizontal axis by the atom directly coordinated to the metal ion: either O, N, C, Cl, or I. L refers to the sum of the UMEs of all these atoms, plus UMEs for Tm-Tm bonds. Polyhedron refers to the sum of all UMEs in L plus the sum of all UMEs between any two atoms of the coordination polyhedron. The top of the light orange bar indicates the UME of the least accurate of the Sparkle models, and the bottom of the top light orange bar indicates the UME of the most accurate of the Sparkle models.

Fig 3 shows the UME_(Tm-L)_ for each type of directly coordinated atom L, indicated on the horizontal axis. The blue bars on the left side of the double bars represent the mean errors of the RM1 model, while the brown bars on the right side of the double bars represent the range of the mean errors of the various Sparkle models. The bottom part of the light brown bar indicates the error of the most accurate Sparkle model, and the top part of the light brown bar indicates the error of the least accurate of the Sparkle models for the particular type of bond.

**Fig 4:**
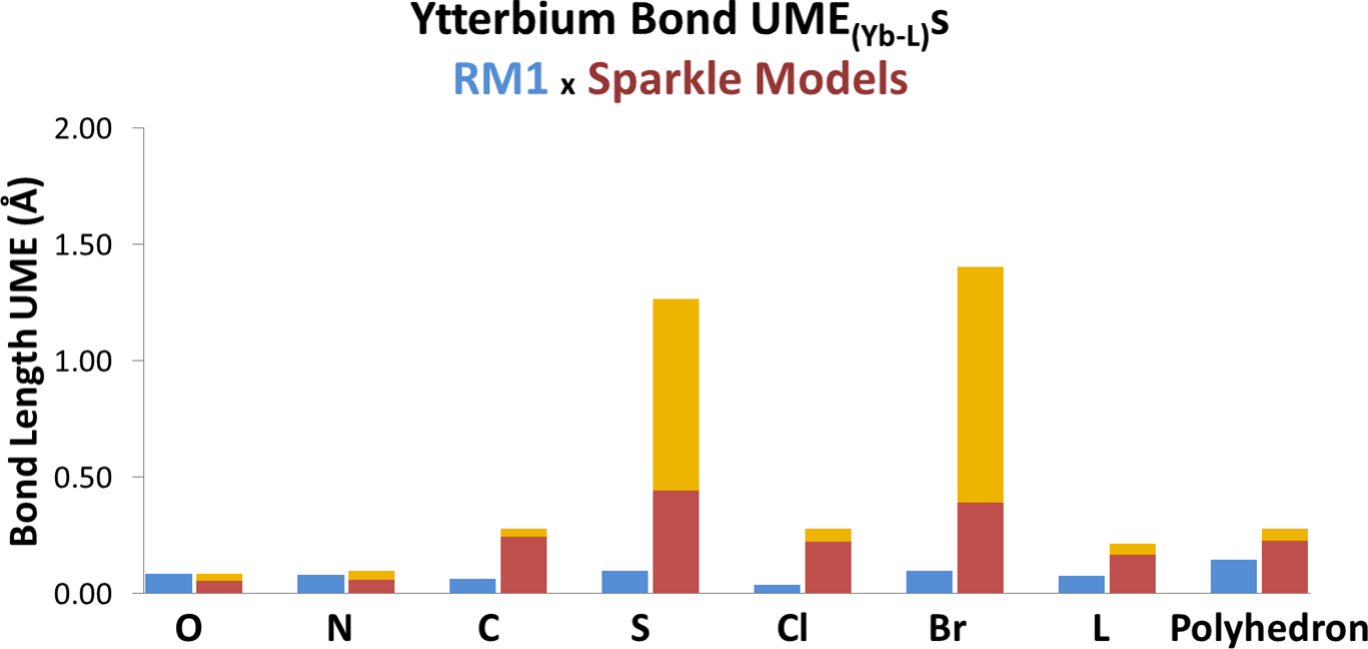
Comparison between the accuracy measures, UME_(Yb-L)_s, of the RM1 model for Yb(III), being advanced in this article (blue bars) and the various Sparkle models (two-lightness bars) for the various types of directly coordinated bonds, indicated on the horizontal axis by the atom directly coordinated to the metal ion: either O, N, C, S, Cl, or Br. L refers to the sum of the UMEs of all these atoms, plus UMEs for Tm-Tm bonds. Polyhedron refers to the sum of all UMEs in L plus the sum of all UMEs between any two atoms of the coordination polyhedron. The top of the light orange bar indicates the UME of the least accurate of the Sparkle models, and the bottom of the top light orange bar indicates the UME of the most accurate of the Sparkle models.

**Fig 5:**
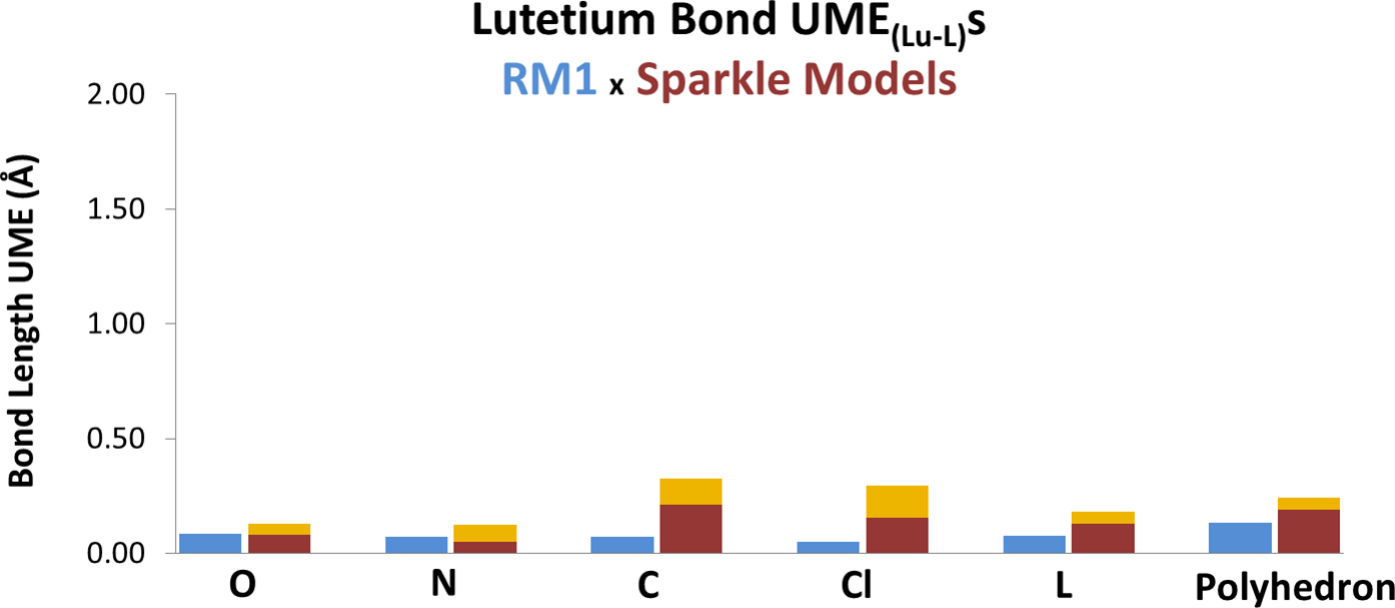
Comparison between the accuracy measures, UME_(Lu-L)_s, of the RM1 model for Lu(III), being advanced in this article (blue bars) and the various Sparkle models (two-lightness bars) for the various types of directly coordinated bonds, indicated on the horizontal axis by the atom directly coordinated to the metal ion: either O, N, C, S, Cl, or Br. L refers to the sum of the UMEs of all these atoms, plus UMEs for Tm-Tm bonds. Polyhedron refers to the sum of all UMEs in L plus the sum of all UMEs between any two atoms of the coordination polyhedron. The top of the light orange bar indicates the UME of the least accurate of the Sparkle models, and the bottom of the top light orange bar indicates the UME of the most accurate of the Sparkle models.

Figs 4 and 5 show corresponding figures for ytterbium and lutetium, respectively. By examining the three figures, one can immediately note that all Sparkle models are indeed accurate for Ln-O and Ln-N bonds, for all three lanthanides. However, the errors significantly increase when the metals are coordinated to a carbon atom or to a chlorine. The errors then become unacceptably large when the directly coordinated atom to the lanthanide is either S, Br or I.

Tables 2–4 show the raw data used to build Figs 3–5. Take the case of Tm-I bonds in Table 2. The most accurate sparkle model is Sparkle/PM6 with an UME_(Tm-I)_ = 0.4345Å, whereas the least accurate is Sparkle/PM7 with a corresponding value of 1.6763Å. This is what is indicated in Fig 3 by the light brown bar over the symbol I.

**Table 2:** RM1, Sparkle/AM1, Sparkle/PM3, Sparkle/PM6, Sparkle/PM7 and Sparkle/RM1 Unsigned Mean Errors (Å) for Thulium.

Type of distances	N	RM1	*AM1	*PM3	*PM6	*PM7	*RM1
Tm–Tm	2	**0.176**	0.278	0.228	0.296	0.362	0.260
Tm–O	20	**0.048**	0.067	0.070	0.066	0.065	0.067
Tm–N	30	**0.051**	0.060	0.089	0.078	0.098	0.067
Tm–C	21	**0.043**	0.236	0.247	0.224	0.327	0.255
Tm–Cl	7	**0.045**	0.218	0.195	0.199	0.129	0.284
Tm–I	3	**0.089**	0.521	0.487	0.435	1.676	0.521
Tm -	83	**0.050**	0.101	0.107	0.100	0.133	0.106
L–L’	04	**0.127**	0.204	0.184	0.225	0.238	0.220
Tm -L, Tm—Tm and L-L’	887	**0.111**	0.183	0.168	0.200	0.216	0.197

*Sparkle Models.

**Table 3:** RM1, Sparkle/AM1, Sparkle/PM3, Sparkle/PM6, Sparkle/PM7 and Sparkle/RM1 Unsigned Mean Errors for Ytterbium.

Type of distances	N	RM1	*AM1	*PM3	*PM6	*PM7	*RM1
Yb—Yb	9	0.228	0.210	**0.207**	0.241	0.529	0.247
Yb—O	231	0.086	0.074	0.074	**0.058**	0.087	0.075
Yb—N	97	0.080	0.069	0.079	0.101	0.072	**0.061**
Yb—C	242	**0.063**	0.280	0.254	0.275	0.247	0.253
Yb—S	21	**0.098**	0.502	0.442	0.538	1.266	0.468
Yb—Cl	29	**0.039**	0.268	0.222	0.276	0.224	0.281
Yb—Br	8	**0.099**	0.450	0.394	0.513	1.403	0.462
Yb—L	637	**0.077**	0.182	0.169	0.183	0.216	0.171
L—L’	2765	**0.164**	0.289	0.244	0.287	0.294	0.274
Yb -L, Yb—Yb and L-L’	3402	**0.148**	0.269	0.230	0.268	0.279	0.255

*Sparkle Models.

**Table 4:** RM1, Sparkle/AM1, Sparkle/PM3, Sparkle/PM6, Sparkle/PM7 and Sparkle/RM1 Unsigned Mean Errors for Lutetium.

Type of distances	N	RM1	*AM1	*PM3	*PM6	*PM7	*RM1
Lu–Lu	5	0.184	0.233	0.222	0.230	**0.144**	0.217
Lu—O	212	0.086	0.083	**0.082**	0.089	0.127	0.090
Lu—N	98	0.073	**0.050**	0.056	0.050	0.126	0.062
Lu—C	130	**0.072**	0.272	0.259	0.246	0.326	0.211
Lu—Cl	30	**0.052**	0.278	0.250	0.296	0.155	0.289
Lu—L	475	**0.078**	0.142	0.137	0.138	0.183	0.131
L—L’	1984	**0.144**	0.231	0.211	0.249	0.258	0.204
Lu -L, Lu-Lu and L-L’	2459	**0.132**	0.214	0.197	0.227	0.243	0.190

*Sparkle Models.

Furthermore, on Table 3, one can see that Yb-C bonds are very common. Actually, bonds of the type Yb-C are more numerous (242 bonds) in the universe set of Yb complexes in CSD than bonds of the types Yb-O (231 bonds) or Yb-N (97 bonds). The same trend occurs for lutetium as one can clearly see from Table 4. The larger errors of the previous sparkle models occur for sulfur, bromine and iodine. However, complexes with these atoms directly coordinated to the lanthanides are rare. So much so, that we could not even find any such case in the universe set of complexes of lutetium.

The RM1 model calculates isolated structures. However, due to its semiempirical character, it is nevertheless able to predict crystallographic structures with high accuracy, implicitly taking into account solid state effects, such as packing effects.

## Conclusion

Results indicate that the present RM1 models for thulium, ytterbium, and lutetium do indeed correct inadequacies of the previous Sparkle models, especially for ytterbium and lutetium, where mainly bonds with carbon atoms directly coordinated to the lanthanide ion are very common.

In conclusion, if the complex of interest has any directly coordinated atoms other than oxygen or nitrogen, then the usage of the present RM1 model for thulium, ytterbium and lutetium is indispensable.

## Supporting Information

S1 File Instructions to run the RM1 calculations. Instructions on how to run the RM1 model for the lanthanides in MOPAC2012, together with sample calculations on complexes of each of the parameterized lanthanide trications: Tm(III), Yb(III), and Lu(III).
(DOCX)
