# Supplementary material for: Parameters for the RM1 Quantum Chemical Calculation of Complexes of the Trications of Thulium, Ytterbium and Lutetium
Source: PLoS One. 2016 May 25;11(5):e0154500. doi: 10.1371/journal.pone.0154500 (PMC4880313; doi:10.1371/journal.pone.0154500)
Supplement: S1 File — Instructions on how to run the RM1 model for the lanthanides in MOPAC2012, together with sample calculations on complexes of each of the parameterized lanthanide trications: Tm(III), Yb(III), and Lu(III). (DOCX) [file pone.0154500.s001.docx]

Parameters for the RM1 quantum chemical calculation of complexes of the trications of thulium, ytterbium, and lutetium

Supporting Information

Manoel A. M. Filho^1^, José Diogo L. Dutra^1^, Gerd B. Rocha^2^, Alfredo M. Simas^3^_,_ and Ricardo O. Freire^1*^

^1^Pople Computational Chemistry Laboratory, Departamento de Química, Universidade Federal de Sergipe, São Cristóvão, SE, Brazil.

^2^Departamento de Química, CCEN, Universidade Federal da Paraíba, João Pessoa, PB, Brazil.

^3^Departamento de Química Fundamental, Universidade Federal de Pernambuco, Recife, PE, Brazil.

^*^Corresponding author. e-mail: [rfreire@ufs.br](mailto:rfreire@ufs.br) (R.O.F.); [simas@ufpe.br](mailto:simas@ufpe.br) (A.M.S)

Contents

| 1. [How to run lanthanide complexes RM1 calculations with MOPAC2012.](#link13) ………………. | 2 |
| --- | --- |
| 1. [MOPAC2012 Input (.mop) and output (.arc) files.](#link3) ……………………………………………….…….. | 4 |
| 1. [Graphical User Interfaces for MOPAC2012](#link4) ………………………………………………………………. | 4 |
| 1. [Additional Tables and Figures](#link4a)..………………………………………………………………….……………… | 5 |
| 1. [Sample Input and Output Files](#link6)……………………………………………………………………..…………… | 11 |
| 1. [References](#link6)………………………………………………………………………………………………………………… | 24 |

1. **How to run lanthanide complexes RM1 calculations with MOPAC2012**

([back to contents](#Contents))

MOPAC2012 is the new software released by Prof. James J. P. Stewart from *Stewart Computational Chemistry* of Colorado Springs, CO, and represents the most recent version of the MOPAC series of molecular modeling softwares, which started in 1981.

MOPAC2012 has Sparkle/AM1, Sparkle/PM3, Sparkle/PM6, Sparkle/PM7, and Sparkle/RM1 fully implemented. Instructions on how to use the Sparkle Model in MOPAC2012, and on how to visualize the complexes with graphical user interfaces, can be found at <http://www.sparkle.pro.br>.

A MOPAC2012 executable can be obtained from <http://openmopac.net> and is presently free for academics.

In order to be acquainted with the software, users are encouraged to read the MOPAC2012 manual at <http://openmopac.net/manual/>.

As the MOPAC2012 manual says:

*MOPAC is written with the non-theoretician in mind.*

*While MOPAC calls upon many concepts in quantum theory and thermodynamics and uses some fairly advanced mathematics, the users need not be familiar with these specialized topics.*

At present, the most recent version of MOPAC2012 is 13.357W.

To run a RM1 model for lanthanides calculation in MOPAC 2012, proceed as follows:

1. Create a data-file with extension .mop which describes a molecular system and specifies the type of calculation that is to be carried out.
   1. Use only the keyword [RM1](http://openmopac.net/manual/rm1_key.html). Do not forget to set the charge n of the complex with keyword [CHARGE=n](http://openmopac.net/manual/charge.html)

To run a Sparkle/RM1 calculation in MOPAC 2012, proceed as follows:

- 1. Use the lanthanide as you would use any atom in MOPAC.
  2. Do not forget to set the charge n of the complex with keyword [CHARGE=n](http://openmopac.net/manual/charge.html)
  3. For a Sparkle/RM1 calculation, use the keywords [RM1](http://openmopac.net/manual/am1.html) [SPARKLE](http://openmopac.net/manual/sparkle.html) in the keyword line.

1. Command MOPAC to run the calculation using that data-file.
2. Get the desired output on the system from the output files created by MOPAC.
3. **MOPAC2012 Input (.mop) and output (.arc) files** ([back to contents](#Contents))

Sample input and output files for all Sparkle Models can be found at <http://www.sparkle.pro.br>.

As examples, we are providing in the appendix of this supplementary material the content of a MOPAC2012 input and the corresponding RM1 output file for one complex for each lanthanide ion.

In order to reproduce the calculation, please [request a password and download](http://openmopac.net/download-c.html) MOPAC2012.exe from <http://openmopac.net>, which is presently free for academics. Then, copy the contents of one of the sample inputs to a text file, name it something like sample.mop, and simply open it with MOPAC2012.

Warning: MOPAC2012 output files with extension .arc may be confused with some types of compressed files on some Windows systems. Be sure to open them with notepad, or a similar text editor.

1. **Graphical User Interfaces for MOPAC2012** ([back to contents](#Contents))

A large number of graphical user interfaces, GUIs, that can be used with MOPAC2012, both commercial and free, can be found [here](http://openmopac.net/resellers.html).

Warning: the bond connection algorithm of some of the Graphical User Interfaces may not work efficiently with some high coordination number lanthanide complexes. Some coordinating bonds may not appear, while sometimes some other spurious bond connections may also appear. However, the positions of the atoms are always correct.

1. **Additional Tables and Figures** ([back to contents](#Contents))

**Table A:** Unsigned mean errors, UME_(Tm-L)_s and UMEs, for RM1 model for the lanthanides, as compared to the respective experimental crystallographic values, obtained from the Cambridge Structural Database,^(1)-(3)^ for each of the 19 thulium (III) complexes.

| **Structure** | **RM1** | | **Structure** | **RM1** | |
| --- | --- | --- | --- | --- | --- |
|  | **UME_(Tm-L)_s (Å)** | **UME (Å)** |  | **UME_(Tm-L)_s (Å)** | **UME (Å)** |
| BAGBIV | 0.0280 | 0.1002 | MIHPAU | 0.0411 | 0.1050 |
| COZHII | 0.0469 | 0.1171 | **NIHZUZ** | 0.0670 | 0.0899 |
| **EWIQAC** | 0.0722 | 0.1333 | PEDROG | 0.0402 | 0.1035 |
| **FAGYUI** | 0.0721 | 0.2207 | **QUDWER** | 0.0193 | 0.0690 |
| FENWOK | 0.0184 | 0.0840 | **SEWWAT** | 0.0358 | 0.1112 |
| **HANCUU** | 0.0418 | 0.1299 | TUPYUY | 0.0356 | 0.0469 |
| **IWUJOZ** | 0.0930 | 0.1609 | TUTXOV | 0.0806 | 0.0918 |
| **KITHAW** | 0.0297 | 0.0840 | **VEQFUS** | 0.0275 | 0.0796 |
| LEYJOO | 0.0212 | 0.1150 | **ZZZARJ01** | 0.0667 | 0.1024 |
| MEDNAK | 0.0530 | 0.1514 |  |  |  |

**Table B:** Unsigned mean errors, UME_(Yb-L)_s and UMEs, for RM1 model for the lanthanides, as compared to the respective experimental crystallographic values, obtained from the Cambridge Structural Database,^(1)-(3)^ for each of the 60 ytterbium (III) complexes.

| **Structure** | **RM1** | | **Structure** | **RM1** | |
| --- | --- | --- | --- | --- | --- |
|  | **UME_(Yb-L)_s (Å)** | **UME (Å)** |  | **UME_(Yb-L)_s (Å)** | **UME (Å)** |
| **AWEJER** | 0.0600 | 0.1560 | HOYKIP | 0.1052 | 0.1646 |
| **BAJQIM** | 0.0328 | 0.1113 | **JADGAW** | 0.1205 | 0.1964 |
| **BEQTAS** | 0.0541 | 0.0917 | **JADGEA** | 0.1137 | 0.2434 |
| **BEXLAS** | 0.0672 | 0.1474 | JAGQAK | 0.0875 | 0.1635 |
| **BEXLOG** | 0.0927 | 0.2033 | JEMROI | 0.1633 | 0.2268 |
| **BUVYEW01** | 0.1025 | 0.1110 | KAGWOF | 0.0707 | 0.1442 |
| **CAQGAC** | 0.0472 | 0.0988 | **KITHEA** | 0.0400 | 0.1042 |
| **CETAYB** | 0.0667 | 0.1299 | **KOLGIB** | 0.0616 | 0.0970 |
| COGLAL | 0.1392 | 0.1924 | LOEAYB10 | 0.0790 | 0.1188 |
| **DALTER** | 0.0831 | 0.1526 | **LORKUY** | 0.0897 | 0.1880 |
| **DANNOV** | 0.0586 | 0.1253 | MIPTUA | 0.0724 | 0.1050 |
| DEKBUR | 0.1107 | 0.1787 | NOSJOU | 0.1338 | 0.1758 |
| DIBKUU | 0.0537 | 0.0833 | **NULYIC** | 0.0652 | 0.1491 |
| DIYNOO | 0.0361 | 0.1208 | PEDJIR02 | 0.0954 | 0.1430 |
| EBUWAZ | 0.1508 | 0.2607 | **PETYES** | 0.0745 | 0.1354 |
| **EBUWUT** | 0.0846 | 0.1717 | **POHFAT** | 0.0018 | 0.0018 |
| ECOJAH | 0.0603 | 0.0932 | QAKXIJ | 0.0551 | 0.2755 |
| **EFIZUO** | 0.0419 | 0.0826 | QALFIS | 0.0576 | 0.1180 |
| EVEQAX | 0.0784 | 0.1299 | **QAXYAP** | 0.0664 | 0.1430 |
| EWIJAV | 0.0582 | 0.0944 | **QOZVUW** | 0.0336 | 0.1129 |
| **FEKXOI** | 0.0802 | 0.0812 | QUCYOC | 0.1113 | 0.1700 |
| FIHMUF | 0.0903 | 0.2024 | QUDWAN | 0.0937 | 0.2922 |
| **FONQUU01** | 0.0558 | 0.1240 | **RAMXEI** | 0.0707 | 0.1096 |
| FOZPOZ | 0.0584 | 0.1361 | RENXIR | 0.0674 | 0.1658 |
| FUTSAO | 0.0514 | 0.1346 | **RIGTOQ** | 0.1052 | 0.1512 |
| GAKYUM | 0.0446 | 0.1259 | **RIHRAB** | 0.0761 | 0.2017 |
| **GEIAYB10** | 0.0472 | 0.0951 | **ROGRIO** | 0.0714 | 0.1346 |
| **GOTZEU** | 0.0277 | 0.1400 | XEFBAM | 0.0902 | 0.1839 |
| HEDVUI | 0.0404 | 0.0863 | **XEWVUQ** | 0.0468 | 0.1350 |
| HOCZAB | 0.0885 | 0.1102 | XOHVEV | 0.0553 | 0.0822 |

**Table C:** Unsigned mean errors, UME_(Lu-L)_s and UMEs, for RM1 model for the lanthanides, as compared to the respective experimental crystallographic values, obtained from the Cambridge Structural Database,^(1)-(3)^ for each of the 47 lutetium (III) complexes.

| **Structure** | **Method RM1** | | **Structure** | **Method RM1** | |
| --- | --- | --- | --- | --- | --- |
|  | **UME_(Lu-L)_s (Å)** | **UME (Å)** |  | **UME_(Lu-L)_s (Å)** | **UME (Å)** |
| **AXOMUV** | 0.0725 | 0.0984 | **NUYPAZ** | 0.0511 | 0.2014 |
| BAGBUH | 0.0730 | 0.0918 | NUYPED | 0.0681 | 0.2068 |
| **BIVCUF** | 0.0449 | 0.1423 | PAGGUZ | 0.1475 | 0.1746 |
| BORQEE | 0.1227 | 0.1937 | POGWEN | 0.3406 | 0.4810 |
| **BUVVUJ** | 0.1044 | 0.1118 | POHDIZ | 0.0464 | 0.1719 |
| DARXEB | 0.0766 | 0.1321 | QIVXOJ | 0.0458 | 0.1643 |
| DICBUM | 0.0217 | 0.0609 | QOFFIB | 0.0764 | 0.1524 |
| **DICCAT** | 0.0330 | 0.0822 | **QQQENA01** | 0.0679 | 0.0936 |
| DIHZID | 0.0630 | 0.1023 | RADRIX | 0.0503 | 0.0749 |
| EFIZEY | 0.0328 | 0.0626 | RADROD | 0.0612 | 0.0812 |
| EFIZOI | 0.0567 | 0.0801 | SIDYIN | 0.0359 | 0.1427 |
| FEWKEX | 0.0575 | 0.1537 | SUDDOK | 0.0518 | 0.1533 |
| **FOPPOP** | 0.0420 | 0.0634 | UFIROQ | 0.1278 | 0.1062 |
| **FUXROF** | 0.0872 | 0.1134 | VEQGAZ | 0.1412 | 0.0809 |
| **HEBQIP** | 0.0610 | 0.0763 | **WAQGAX** | 0.1315 | 0.1665 |
| HELGUA | 0.0529 | 0.1732 | WEFRAA | 0.0654 | 0.1428 |
| **HIZHED** | 0.0721 | 0.1825 | XAWVAS | 0.0676 | 0.0874 |
| HOCZEF | 0.0863 | 0.2022 | XECQUR | 0.0654 | 0.1367 |
| IFUHEX | 0.0692 | 0.1589 | XECRIG | 0.1091 | 0.1489 |
| **IFUHOH** | 0.0826 | 0.1370 | **XEPLUZ** | 0.0231 | 0.0506 |
| **JENNEV** | 0.0621 | 0.1845 | XEWRAS | 0.1133 | 0.1059 |
| KESTUY | 0.0798 | 0.0830 | XEWREW | 0.0699 | 0.1171 |
| NIJHAP | 0.0769 | 0.2419 | XEWWAX | 0.0825 | 0.1286 |
| NITXIY | 0.0902 | 0.2284 |  |  |  |

**Table D.** Means and Variances of the *γ* Distribution Fits for the UME_(Ln-L)_s Computed for the *N* Complexes for Each Lanthanide Trication.

| UME_(Ln-L)_s | | | | |
| --- | --- | --- | --- | --- |
| Lanthanide ion | N | Mean (Å) | Variance (Å^2^) | p-value |
| Tm^3+^ | 19 | 0.0499 | 0.0021 | 0.7360 |
| Yb^3+^ | 60 | 0.0768 | 0.0037 | 0.5409 |
| Lu^3+^ | 47 | 0.0783 | 0.0046 | 0.3856 |

**Table E**. Means and Variances of the *γ* Distribution Fits for the UMEs Computed for the *N* Complexes for Each Lanthanide Trication.

| UMEs | | | | |
| --- | --- | --- | --- | --- |
| Lanthanide ion | N | Mean (Å) | Variance (Å^2^) | p-value |
| Tm^3+^ | 19 | 0.1108 | 0.0165 | 0.9445 |
| Yb^3+^ | 60 | 0.1477 | 0.0202 | 0.1593 |
| Lu^3+^ | 47 | 0.1315 | 0.0204 | 0.9770 |


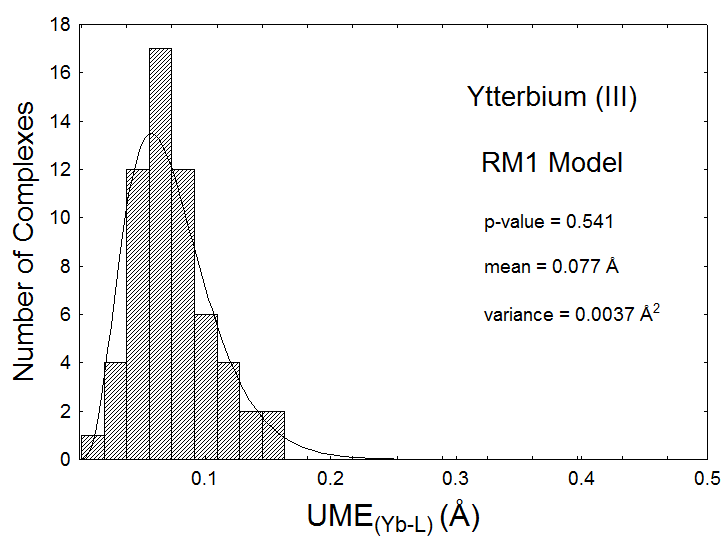


**Figure A:** Histogram of the UME_(Yb-L)_s for all 60 complexes of Yb(III) optimized via the RM1 model being advanced in this article. The mean and variance were obtained from the fitted gamma distribution. The p-value of the one-sample nonparametric Kolmogorov-Smirnoff is also shown. This value is above 0.05, and therefore the data can be considered adjusted for the fitted gamma distribution within a 95% confidence interval

**
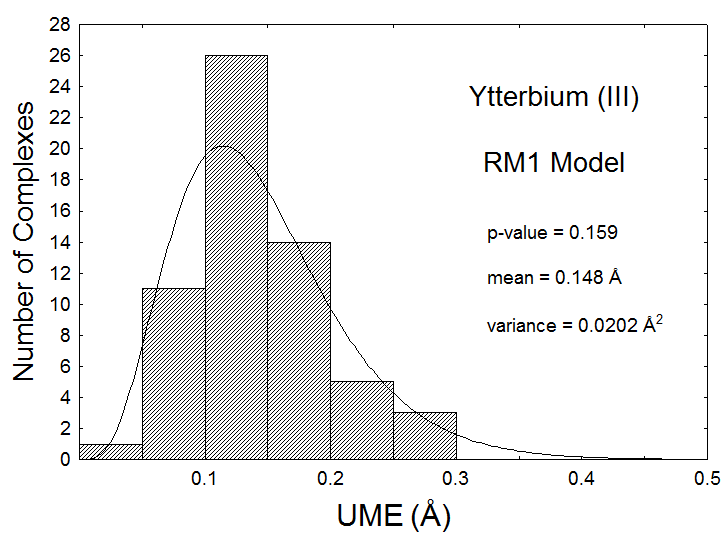
**

**Figure B:** Histogram of the UMEs for all 60 complexes of Yb(III) optimized via the RM1 model being advanced in this article. The mean and variance were obtained from the fitted gamma distribution. The p-value of the one-sample nonparametric Kolmogorov-Smirnoff is also shown. This value is above 0.05, and therefore the data can be considered adjusted for the fitted gamma distribution within a 95% confidence interval.

**
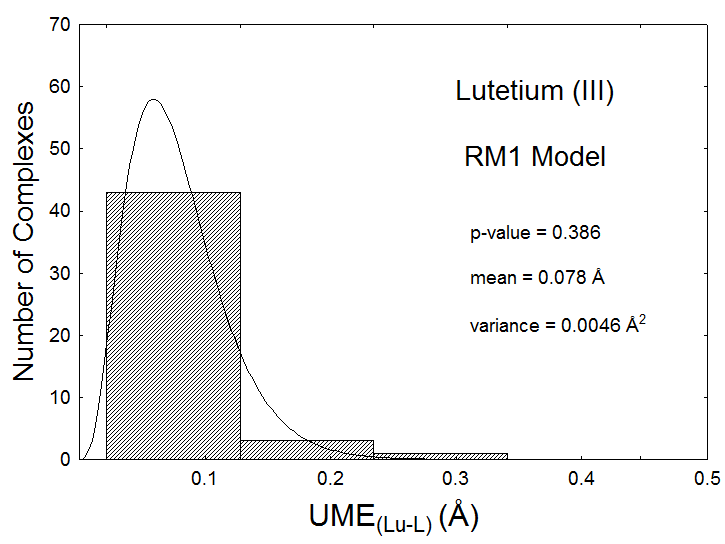
**

**Figure C:** Histogram of the UME_(Lu-L)_s for all 47 complexes of Lu(III) optimized via the RM1 model being advanced in this article. The mean and variance were obtained from the fitted gamma distribution. The p-value of the one-sample nonparametric Kolmogorov-Smirnoff is also shown. This value is above 0.05, and therefore the data can be considered adjusted for the fitted gamma distribution within a 95% confidence interval

**
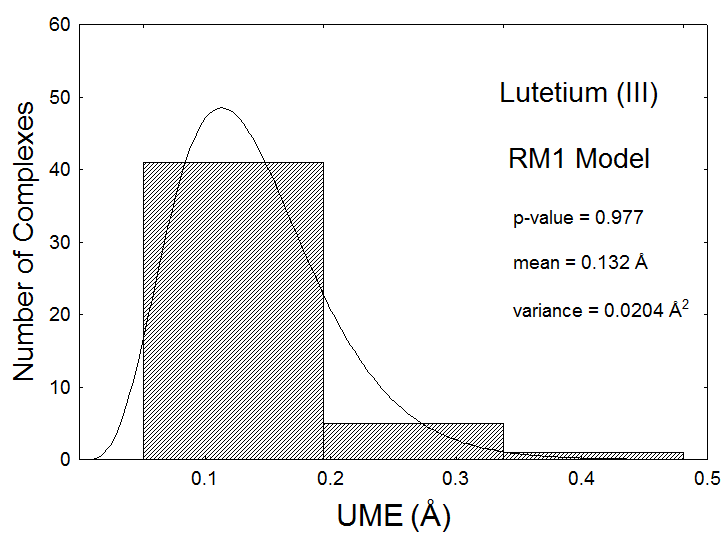
**

**Figure D:** Histogram of the UMEs for all 47 complexes of Lu(III) optimized via the RM1 model being advanced in this article. The mean and variance were obtained from the fitted gamma distribution. The p-value of the one-sample nonparametric Kolmogorov-Smirnoff is also shown. This value is above 0.05, and therefore the data can be considered adjusted for the fitted gamma distribution within a 95% confidence interval.


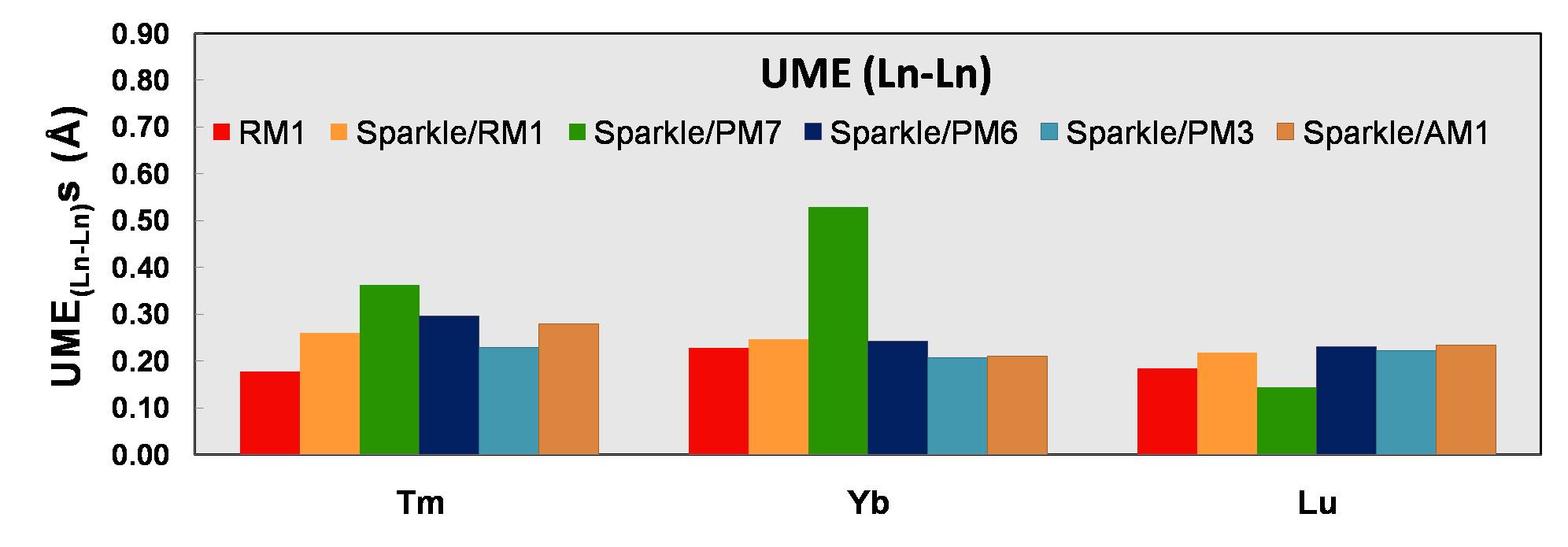


**Figure E:** UME_(Ln-Ln)_s obtained using the RM1 model for lanthanides and all five versions of the Sparkle Model: Sparkle/AM1, Sparkle/PM3, Sparkle/PM6, Sparkle/PM7 and Sparkle/RM1 for all complexes of the validation set, for the lanthanide trications, from Tm(III), Yb(III) and Lu(III). The UME_(Ln-Ln)_s are calculated as the absolute value of the difference between the experimental and calculated interatomic distances between the two lanthanide ions, all summed for all complexes, for each of the lanthanides.


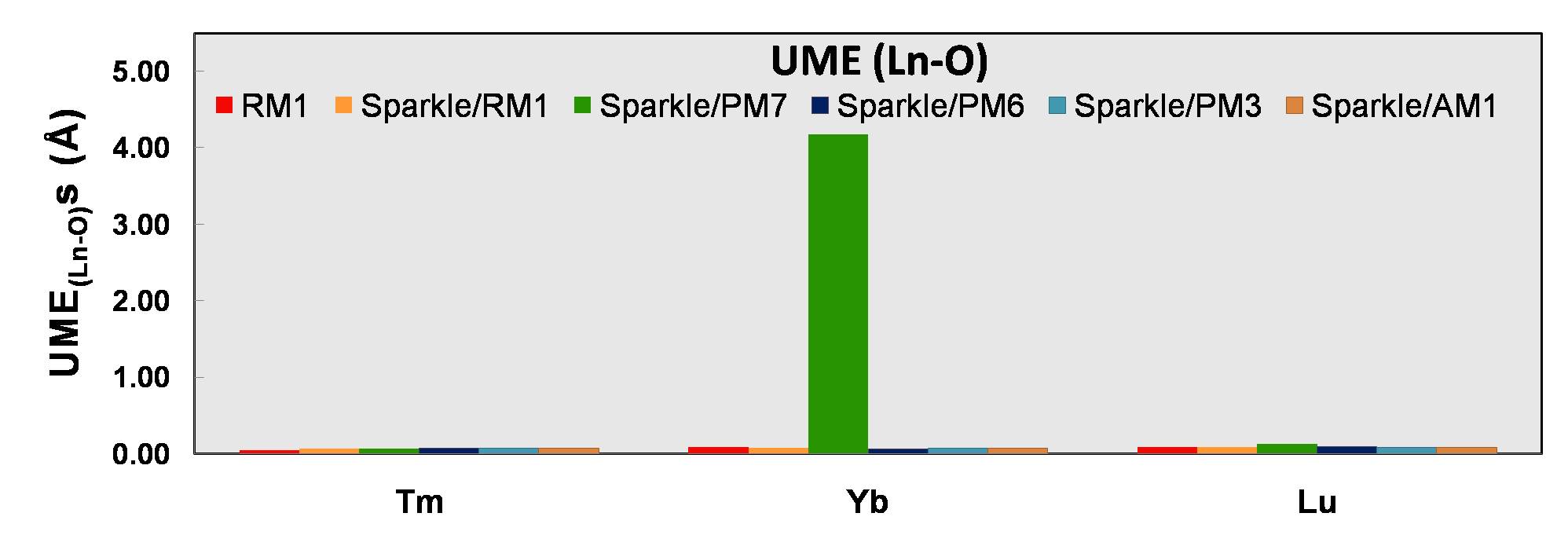


**Figure F:** UME_(Ln-O)_s obtained using the RM1 model for lanthanides and all five versions of the Sparkle Model: Sparkle/AM1, Sparkle/PM3, Sparkle/PM6, Sparkle/PM7 and Sparkle/RM1 for all complexes of the validation set, for the lanthanide trications, from Tm(III), Yb(III) and Lu(III). The UME_(Ln-O)_s are calculated as the absolute value of the difference between the experimental and calculated interatomic distances between the lanthanide ion and the directly coordinating oxygen atoms, summed for all complexes, for each of the lanthanides.


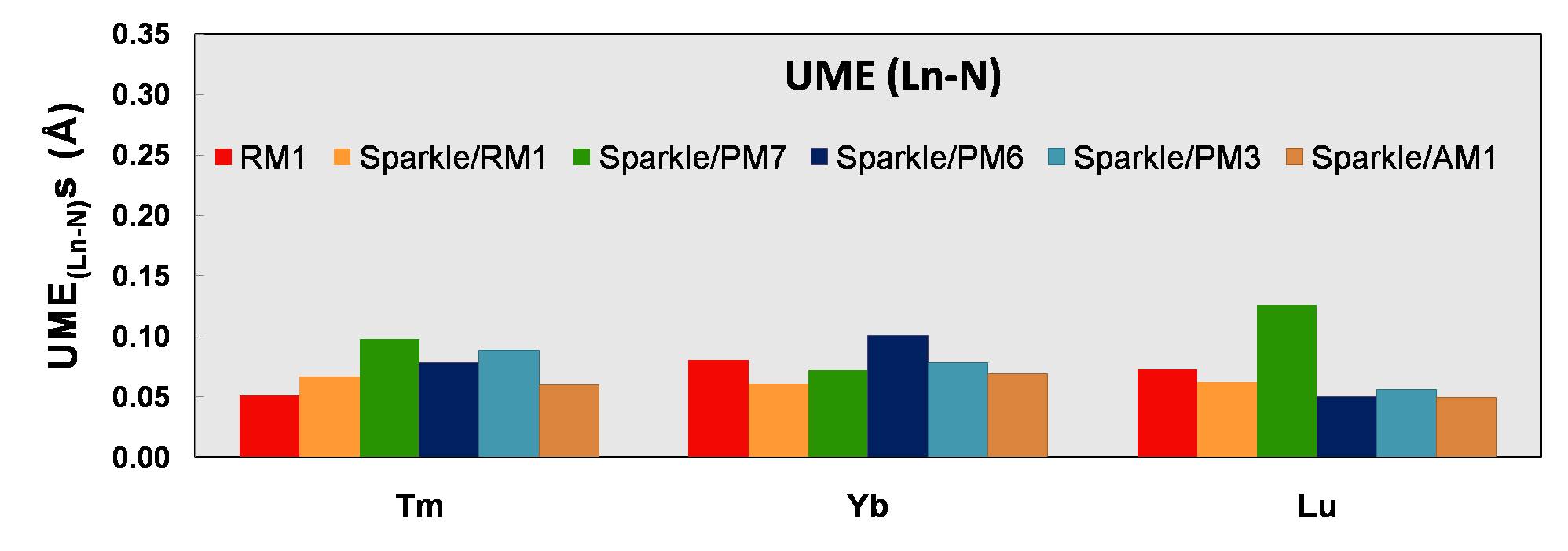


**Figure G:** UME_(Ln-N)_s obtained using the RM1 model for lanthanides and all five versions of the Sparkle Model: Sparkle/AM1, Sparkle/PM3, Sparkle/PM6, Sparkle/PM7 and Sparkle/RM1 for all complexes of the validation set, for the lanthanide trications, from Tm(III), Yb(III) and Lu(III). The UME_(Ln-N)_s are calculated as the absolute value of the difference between the experimental and calculated interatomic distances between the lanthanide ion and the directly coordinating nitrogen atoms, summed for all complexes, for each of the lanthanides.

**
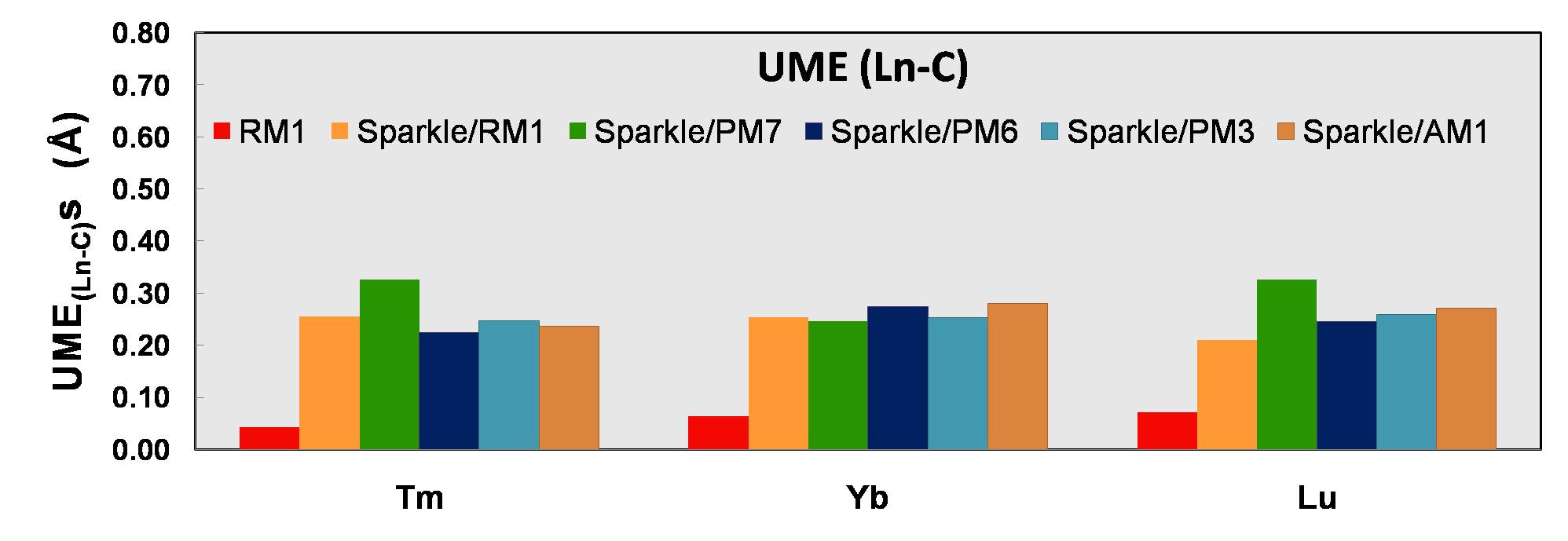
**

**Figure H:** UME_(Ln-C)_ obtained using the RM1 model for lanthanides and all five versions of the Sparkle Model: Sparkle/AM1, Sparkle/PM3, Sparkle/PM6, Sparkle/PM7 and Sparkle/RM1 for all complexes of the validation set, for the lanthanide trications, from Tm(III), Yb(III) and Lu(III). The UME_(Ln-C)_ are calculated as the absolute value of the difference between the experimental and calculated interatomic distances between the lanthanide ion and the directly coordinating carbon atoms, summed for all complexes, for each of the lanthanides.


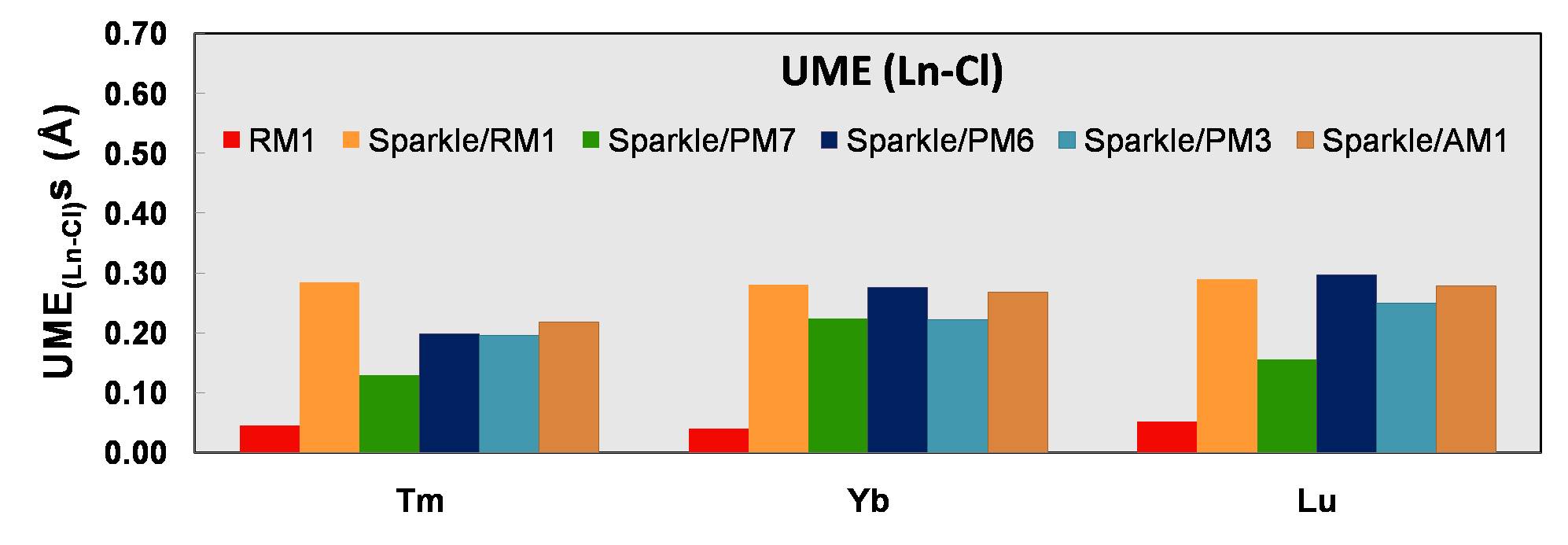


**Figure I:** UME_(Ln-Cl)_s obtained using the RM1 model for lanthanides and all five versions of the Sparkle Model: Sparkle/AM1, Sparkle/PM3, Sparkle/PM6, Sparkle/PM7 and Sparkle/RM1 for all complexes of the validation set, for the lanthanide trications, Tm(III), Yb(III) and Lu(III). The UME_(Ln-Cl)_s are calculated as the absolute value of the difference between the experimental and calculated interatomic distances between the lanthanide ion and the directly coordinating chlorine atoms, summed for all complexes, for each of the lanthanides.


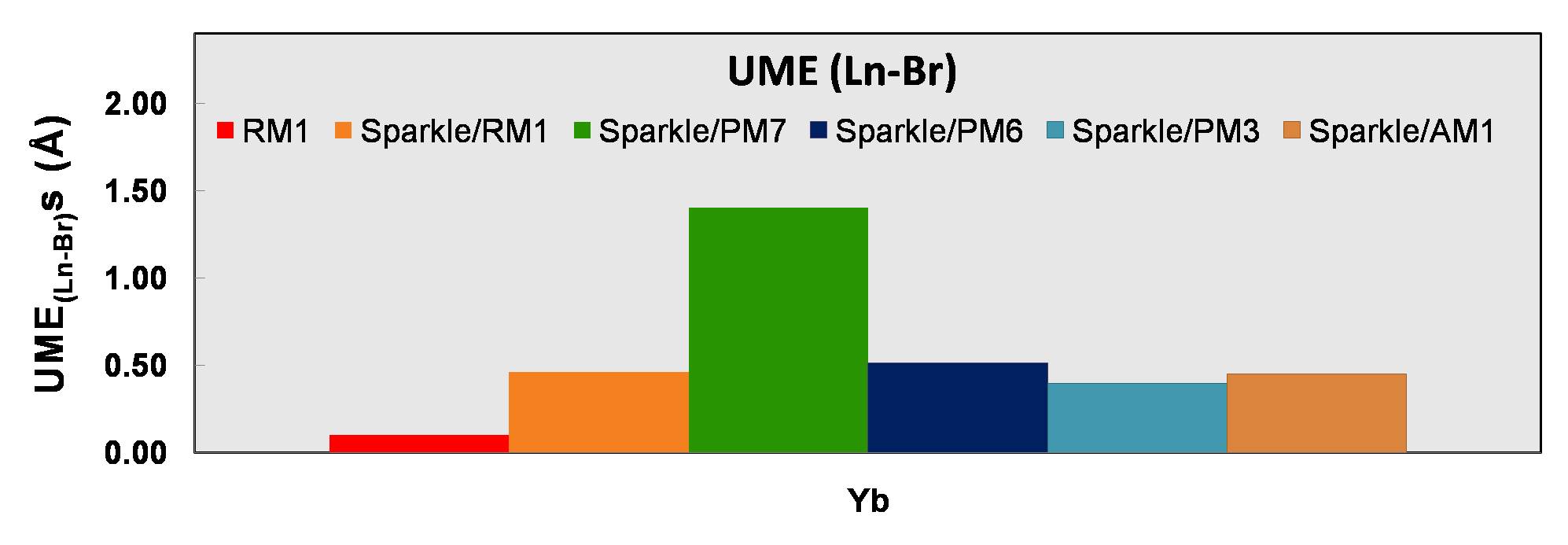


**Figure J:** UME_(Ln-Br)_s obtained using the RM1 model for lanthanides and all five versions of the Sparkle Model: Sparkle/AM1, Sparkle/PM3, Sparkle/PM6, Sparkle/PM7 and Sparkle/RM1 for all complexes of the validation set, for the lanthanide trications Yb (III). The UME_(Ln-Br)_s are calculated as the absolute value of the difference between the experimental and calculated interatomic distances between the lanthanide ion and the directly coordinating chlorine atoms, summed for all complexes, for each of the lanthanides.


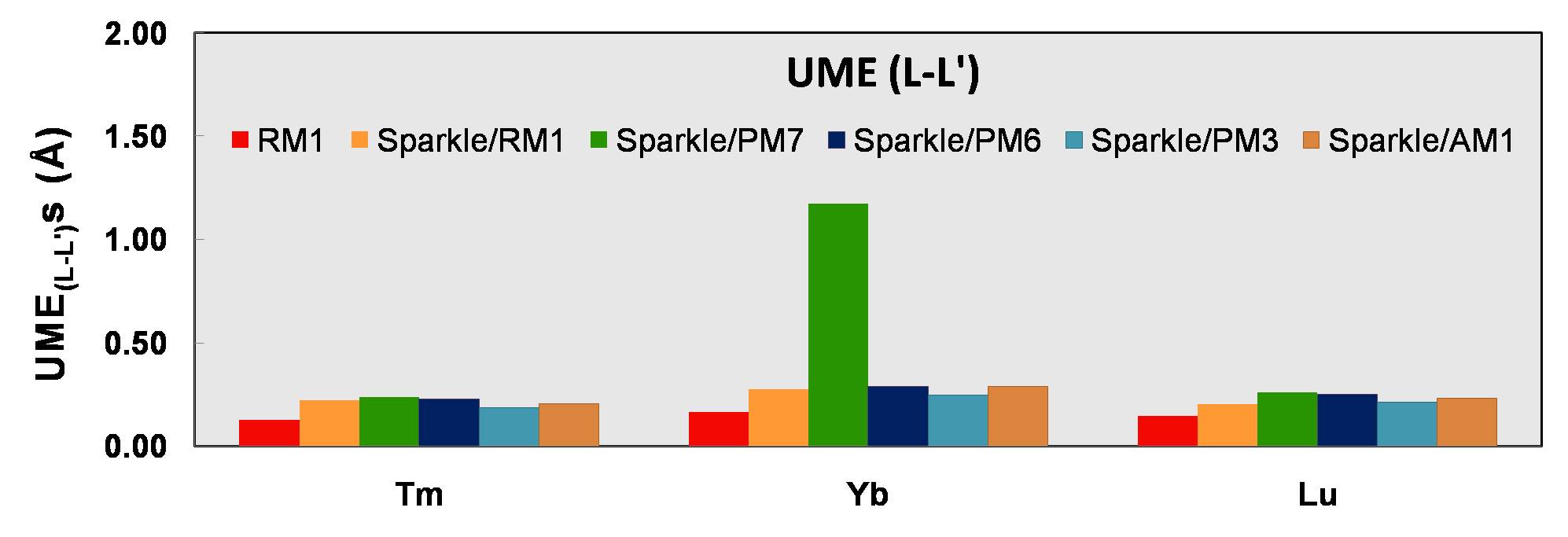


**Figure K:** UME_(L-L)_s obtained using the RM1 model for lanthanides and all five versions of the Sparkle Model: Sparkle/AM1, Sparkle/PM3, Sparkle/PM6, Sparkle/PM7 and Sparkle/RM1 for all complexes of the validation set, for the lanthanide trications, from Tm(III), Yb(III) and Lu(III). The UME_(L-L)_s are calculated as the average of the absolute value of the difference between the experimental and calculated interatomic distances, summed over all interatomic distances between all atoms of the coordination polyhedra, for all complexes, for each of the lanthanides.


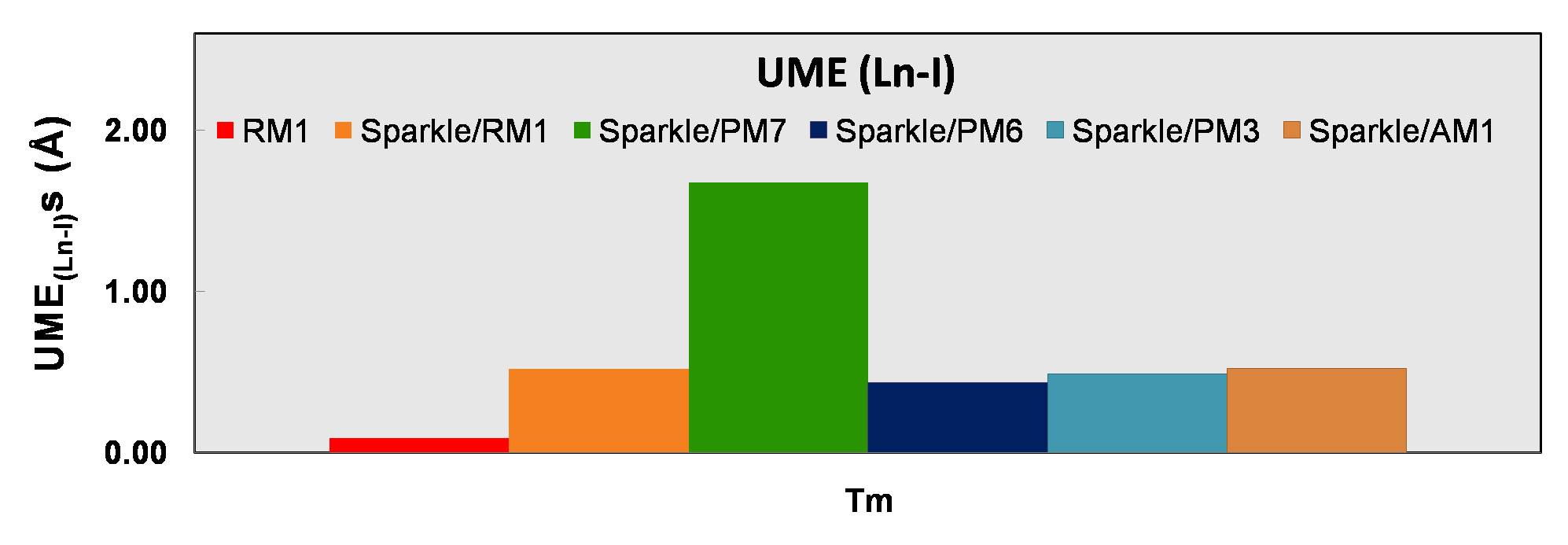


**Figure L:** UME_(Ln-I)_s obtained using the RM1 model for lanthanides and all five versions of the Sparkle Model: Sparkle/AM1, Sparkle/PM3, Sparkle/PM6, Sparkle/PM7 and Sparkle/RM1 for all complexes of the validation set, for the lanthanide trications, from Tm(III). The UME_(Ln-I)_s are calculated as the average of the absolute value of the difference between the experimental and calculated interatomic distances, summed over all interatomic distances between all atoms of the coordination polyhedra, for all complexes, for each of the lanthanides.

1. **Sample Input and Output Files** ([back to contents](#Contents))

**Thulium: KITHAW**

**
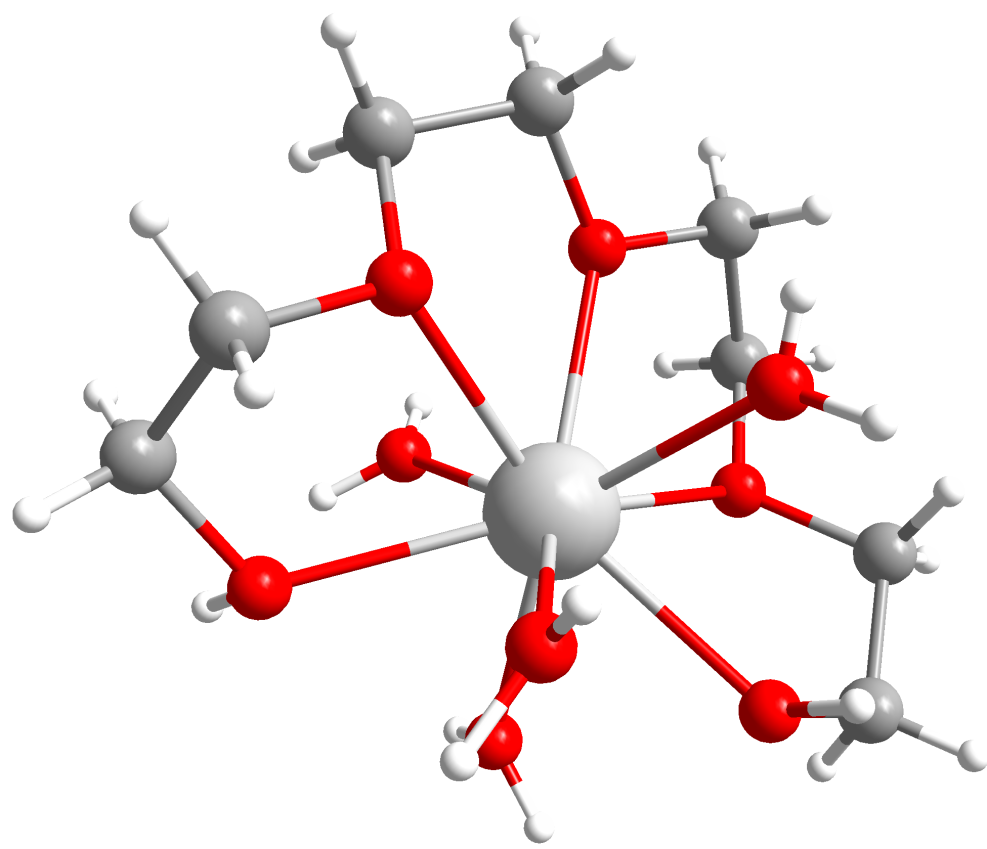
**

---------------------------------------- Begin of file **KITHAW.mop**-----------------------------------

RM1 PRECISE NOINTER XYZ BFGS T=10D GNORM=0.25 +

NOLOG GEO-OK SCFCRT=1.D-10 CHARGE=3.0

NUMERO DE COORDENAÇÃO= 9

Tm 0.0000000 1 0.0000000 1 0.0000000 1

O 2.3948000 1 0.0000000 1 0.0000000 1

O 0.9616788 1 2.2456479 1 0.0000000 1

O -1.3196306 1 1.6591997 1 1.2182551 1

O -1.8868308 1 -0.8722976 1 1.2261590 1

O -1.2396761 1 -1.6760326 1 -1.2642979 1

O 0.8710311 1 -0.0966739 1 -2.1705538 1

O 0.7876536 1 -2.1076228 1 0.5753865 1

O -1.5291225 1 1.0747048 1 -1.4232127 1

O 0.7569520 1 0.0801051 1 2.2119971 1

C 3.0940965 1 1.2303301 1 -0.1546263 1

C 2.3247025 1 2.3287460 1 0.4807247 1

C 0.1299022 1 3.3290098 1 0.4322238 1

C -0.6994331 1 2.9103379 1 1.5974877 1

C -2.3584591 1 1.2589435 1 2.1050544 1

C -2.9662770 1 0.0209109 1 1.5655481 1

C -2.3496892 1 -2.1842324 1 0.8205974 1

C -2.4648037 1 -2.1766036 1 -0.6691658 1

H 2.8086983 1 -0.3100790 1 0.8054632 1

H -1.6157090 1 -1.2949446 1 -2.1885321 1

H 0.5404609 1 0.4188660 1 -2.8475533 1

H 1.6712356 1 -0.2742446 1 -2.3811841 1

H 0.8290924 1 -2.3780883 1 1.2878509 1

H 0.9117313 1 -2.8325753 1 -0.0639091 1

H -1.8371126 1 0.6783361 1 -2.1165885 1

H -1.8460946 1 1.7716389 1 -1.3993877 1

H 0.4089999 1 0.2803097 1 2.7781098 1

H 1.4823672 1 -0.2891258 1 2.7158469 1

H 3.9492268 1 1.1632738 1 0.2497603 1

H 3.1867694 1 1.4257896 1 -1.0796707 1

H 2.6922637 1 3.1744005 1 0.2558732 1

H 2.3384529 1 2.2213733 1 1.4224233 1

H 0.6727070 1 4.0589460 1 0.6842223 1

H -0.4358410 1 3.5947582 1 -0.2782662 1

H -1.3618923 1 3.5635827 1 1.7901251 1

H -0.1518569 1 2.7703448 1 2.3590813 1

H -1.9853382 1 1.0820302 1 2.9619464 1

H -3.0139845 1 1.9434889 1 2.1780234 1

H -3.4889965 1 0.2310147 1 0.8019828 1

H -3.5135825 1 -0.3849919 1 2.2325278 1

H -1.7367040 1 -2.8439638 1 1.1164317 1

H -3.2088778 1 -2.3525574 1 1.1921520 1

H -3.2047854 1 -1.6505778 1 -0.9588777 1

H -2.5894169 1 -3.0742075 1 -0.9545930 1

---------------------------------------- End of file **KITHAW.mop**-----------------------------------

---------------------------------------- Begin of file **KITHAW.arc**-----------------------------------

SUMMARY OF RM1 CALCULATION, Site No: 3560

MOPAC2012 (Version: 13.357W)

Thu Feb 05 12:50:20 2015

No. of days left = 0

Empirical Formula: C8 H26 O9 Tm = 44 atoms

RM1 PRECISE NOINTER XYZ BFGS T=10D GNORM=0.25 +

NOLOG GEO-OK SCFCRT=1.D-10 CHARGE=3.0

COORDINATION NUMBER = 9

PETERS TEST WAS SATISFIED IN BFGS OPTIMIZATION

SCF FIELD WAS ACHIEVED

HEAT OF FORMATION = 572.24351 KCAL/MOL = 2394.26684 KJ/MOL

TOTAL ENERGY = -4238.39773 EV

ELECTRONIC ENERGY = -29314.48737 EV

CORE-CORE REPULSION = 25076.08964 EV

GRADIENT NORM = 0.23550

DIPOLE = 1.71714 DEBYE POINT GROUP: C1

NO. OF FILLED LEVELS = 56

CHARGE ON SYSTEM = 3

IONIZATION POTENTIAL = 20.691377 EV

HOMO LUMO ENERGIES (EV) = -20.691-10.628

MOLECULAR WEIGHT = 435.222

COSMO AREA = 272.23 SQUARE ANGSTROMS

COSMO VOLUME = 352.99 CUBIC ANGSTROMS

MOLECULAR DIMENSIONS (Angstroms)

Atom Atom Distance

H 29 H 40 8.27227

H 35 H 44 7.34603

H 25 H 27 5.64367

SCF CALCULATIONS = 335

WALL-CLOCK TIME = 12.016 SECONDS

COMPUTATION TIME = 23.891 SECONDS

FINAL GEOMETRY OBTAINED CHARGE

RM1 PRECISE NOINTER XYZ BFGS T=10D GNORM=0.25 +

NOLOG GEO-OK SCFCRT=1.D-10 CHARGE=3.0

COORDINATION NUMBER = 9

Tm 0.06303009 +1 0.01157496 +1 0.07678012 +1 0.1848

O 2.41586541 +1 0.15822622 +1 0.28598738 +1 -0.2670

O 1.02784789 +1 2.24517301 +1 0.08688193 +1 -0.2400

O -1.26068642 +1 1.70512114 +1 1.25054204 +1 -0.2485

O -1.88680958 +1 -0.85447959 +1 1.30250719 +1 -0.2352

O -1.25404105 +1 -1.59903985 +1 -1.08950442 +1 -0.2737

O 0.90553573 +1 -0.18266584 +1 -2.06868973 +1 -0.2708

O 0.95543887 +1 -2.10628052 +1 0.30680268 +1 -0.2663

O -1.36378846 +1 1.09364133 +1 -1.36923417 +1 -0.2601

O 0.65927216 +1 -0.31579811 +1 2.27597376 +1 -0.2648

C 3.19146196 +1 1.29901413 +1 -0.09557445 +1 0.0239

C 2.40474151 +1 2.52265551 +1 0.39545238 +1 0.0162

C 0.16078495 +1 3.36203408 +1 0.31657067 +1 0.0192

C -0.74263549 +1 3.02194486 +1 1.51139692 +1 -0.0014

C -2.25273125 +1 1.31007052 +1 2.21267830 +1 0.0181

C -2.94069866 +1 0.06591136 +1 1.63223629 +1 0.0119

C -2.35768960 +1 -2.15041863 +1 0.92005730 +1 0.0176

C -2.46578864 +1 -2.19824788 +1 -0.61202245 +1 -0.0008

H 3.02103529 +1 -0.57533247 +1 0.48280024 +1 0.2866

H -1.16800446 +1 -1.79534190 +1 -2.03743655 +1 0.2840

H 0.94201595 +1 0.54772697 +1 -2.70464724 +1 0.3002

H 1.59572951 +1 -0.80374290 +1 -2.34147635 +1 0.2969

H 1.33222163 +1 -2.49359548 +1 1.11030057 +1 0.2967

H 0.82815638 +1 -2.84558311 +1 -0.30525203 +1 0.2959

H -1.58647187 +1 0.83554870 +1 -2.27406394 +1 0.2907

H -1.98016635 +1 1.80447208 +1 -1.13981231 +1 0.2992

H 0.05473987 +1 -0.60139567 +1 2.97663981 +1 0.3001

H 1.44589030 +1 0.02825610 +1 2.72110580 +1 0.2968

H 4.21531729 +1 1.27918021 +1 0.35717095 +1 0.1478

H 3.33044672 +1 1.28292190 +1 -1.20474897 +1 0.1139

H 2.76039741 +1 3.44359355 +1 -0.13203598 +1 0.1438

H 2.52141046 +1 2.70372652 +1 1.49102986 +1 0.1128

H 0.69734404 +1 4.32637729 +1 0.50826198 +1 0.1516

H -0.39412230 +1 3.52016914 +1 -0.63941062 +1 0.1195

H -1.57168409 +1 3.76953532 +1 1.59231293 +1 0.1424

H -0.19662337 +1 3.03798117 +1 2.48546869 +1 0.1144

H -1.77501436 +1 1.12266613 +1 3.20461729 +1 0.1078

H -3.01225499 +1 2.11733062 +1 2.37432203 +1 0.1442

H -3.56082512 +1 0.29414233 +1 0.73210576 +1 0.1055

H -3.62876367 +1 -0.37336938 +1 2.39921320 +1 0.1458

H -1.59387924 +1 -2.85617270 +1 1.32946932 +1 0.1226

H -3.32869491 +1 -2.43662004 +1 1.39663907 +1 0.1536

H -3.34766706 +1 -1.63783727 +1 -1.00876832 +1 0.1193

H -2.56402795 +1 -3.25225677 +1 -0.97613912 +1 0.1449

---------------------------------------- End of file **KITHAW.arc**-----------------------------------

**Ytterbium: KOLGIB**


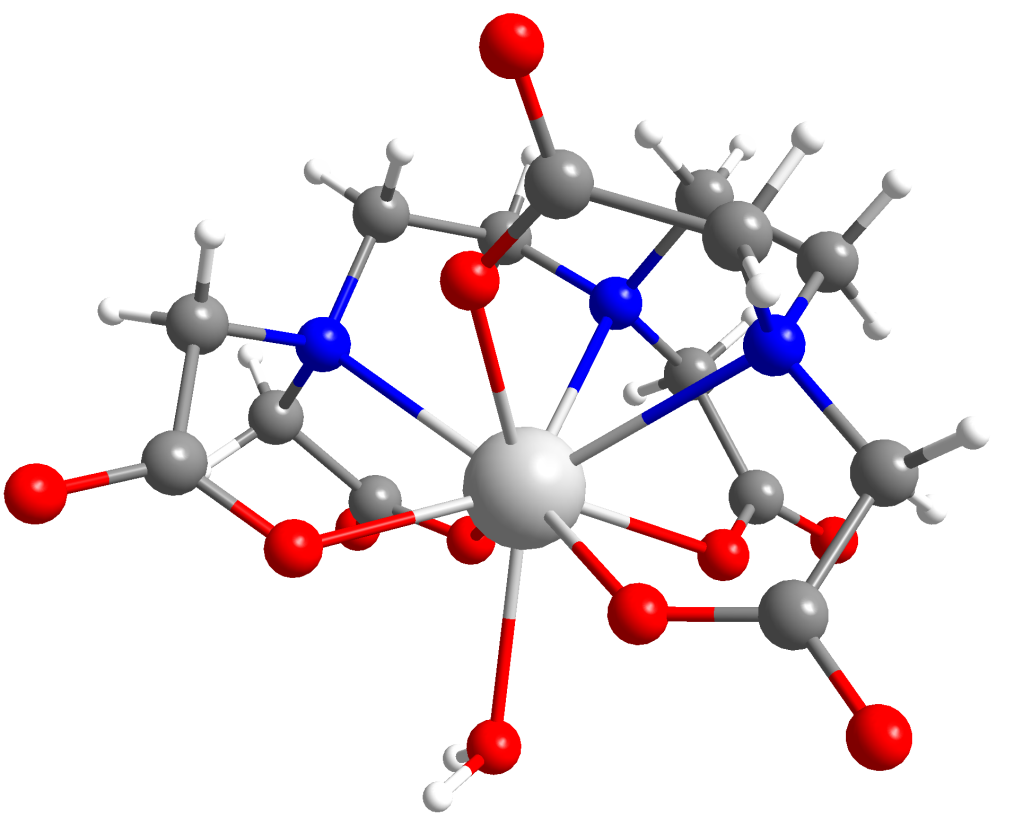


---------------------------------------- Begin of file **KOLGIB.mop**-----------------------------------

RM1 PRECISE NOINTER XYZ BFGS T=10D GNORM=0.25 +

NOLOG GEO-OK SCFCRT=1.D-10 CHARGE=-2.0

COORDINATION NUMBER = 9

Yb 0.240152 1 -0.385442 1 0.103605 1

O 2.535652 1 -0.385442 1 0.103605 1

O -0.114069 1 -0.228020 1 2.394948 1

O -1.750141 1 -1.433307 1 0.537216 1

O 0.871811 1 1.086105 1 -1.574934 1

O 0.459994 1 -2.037017 1 -1.485714 1

O 0.925478 1 -2.431234 1 1.206763 1

N 1.247432 1 1.743361 1 1.088679 1

N -1.631465 1 1.312221 1 0.465913 1

N -1.421947 1 -0.239393 1 -2.072232 1

H 0.954482 1 -2.336148 1 2.100350 1

H 1.722048 1 -2.723116 1 0.907495 1

C 3.269446 1 0.648821 1 0.223682 1

C 2.585219 1 1.974553 1 0.493874 1

O 0.775583 1 2.219318 1 -3.474504 1

O 4.506830 1 0.621334 1 0.103605 1

O 0.244589 1 0.681469 1 4.400775 1

O -3.732548 1 -1.453993 1 1.554982 1

O -0.505661 1 -3.662657 1 -2.627855 1

H 3.125768 1 2.502097 1 1.103119 1

H 2.492416 1 2.468599 1 -0.334304 1

C 1.414396 1 1.589379 1 2.546391 1

H 1.309838 1 2.457678 1 2.967099 1

H 2.316033 1 1.281254 1 2.728078 1

C 0.434672 1 0.622642 1 3.173399 1

C 0.326790 1 2.852615 1 0.779589 1

H 0.361363 1 3.043646 1 -0.171296 1

H 0.612643 1 3.649245 1 1.254294 1

C -1.099360 1 2.516921 1 1.173435 1

H -1.134810 1 2.361035 1 2.130474 1

H -1.669370 1 3.275839 1 0.976284 1

C -2.666772 1 0.642288 1 1.283921 1

H -3.531274 1 1.017166 1 1.057093 1

H -2.497740 1 0.838472 1 2.218300 1

C -2.731027 1 -0.858561 1 1.109534 1

C -2.221344 1 1.727022 1 -0.824230 1

H -1.584514 1 2.281107 1 -1.303293 1

H -3.012110 1 2.261957 1 -0.655516 1

C -2.601402 1 0.529505 1 -1.698915 1

H -3.218437 1 -0.041388 1 -1.215123 1

H -3.048015 1 0.843408 1 -2.500727 1

C -0.710353 1 0.399468 1 -3.189835 1

H -1.344439 1 0.906432 1 -3.721983 1

H -0.327859 1 -0.287253 1 -3.758688 1

C 0.387389 1 1.320124 1 -2.723666 1

C -1.708705 1 -1.647039 1 -2.413340 1

H -1.978568 1 -1.705563 1 -3.343726 1

H -2.442243 1 -1.969418 1 -1.866628 1

C -0.486553 1 -2.513760 1 -2.184561 1

0

---------------------------------------- End of file **KOLGIB.mop**-----------------------------------

---------------------------------------- Begin of file **KOLGIB.arc**-----------------------------------

SUMMARY OF RM1 CALCULATION, Site No: 3560

MOPAC2012 (Version: 13.357W)

Thu Feb 05 12:51:20 2015

No. of days left = 0

Empirical Formula: C14 H20 N3 O11 Yb = 49 atoms

RM1 PRECISE NOINTER XYZ BFGS T=10D GNORM=0.25 +

NOLOG GEO-OK SCFCRT=1.D-10 CHARGE=-2.0

COORDINATION NUMBER = 9

PETERS TEST WAS SATISFIED IN BFGS OPTIMIZATION

SCF FIELD WAS ACHIEVED

HEAT OF FORMATION = -438.53223 KCAL/MOL = -1834.81886 KJ/MOL

TOTAL ENERGY = -6205.77434 EV

ELECTRONIC ENERGY = -50355.78044 EV

CORE-CORE REPULSION = 44150.00610 EV

GRADIENT NORM = 0.22661

DIPOLE = 12.52715 DEBYE POINT GROUP: C1

NO. OF FILLED LEVELS = 81

CHARGE ON SYSTEM = -2

IONIZATION POTENTIAL = 4.853980 EV

HOMO LUMO ENERGIES (EV) = -4.854 3.366

MOLECULAR WEIGHT = 579.365

COSMO AREA = 334.32 SQUARE ANGSTROMS

COSMO VOLUME = 458.74 CUBIC ANGSTROMS

MOLECULAR DIMENSIONS (Angstroms)

Atom Atom Distance

H 28 O 19 10.27040

H 28 O 19 7.37613

O 18 O 16 1.52387

SCF CALCULATIONS = 118

WALL-CLOCK TIME = 9.590 SECONDS

COMPUTATION TIME = 18.766 SECONDS

FINAL GEOMETRY OBTAINED CHARGE

RM1 PRECISE NOINTER XYZ BFGS T=10D GNORM=0.25 +

NOLOG GEO-OK SCFCRT=1.D-10 CHARGE=-2.0

COORDINATION NUMBER = 9

Yb 0.30180729 +1 -0.51134650 +1 0.09873532 +1 -1.0843

O 2.64041306 +1 -0.46488565 +1 0.40197785 +1 -0.3439

O 0.07358779 +1 -0.34860778 +1 2.45522636 +1 -0.3234

O -1.77072874 +1 -1.50458606 +1 0.62214075 +1 -0.3059

O 0.96801104 +1 1.02568765 +1 -1.57572914 +1 -0.3342

O 0.41510584 +1 -2.27373272 +1 -1.44218749 +1 -0.3109

O 1.10653528 +1 -2.60743986 +1 1.07259268 +1 -0.1615

N 1.28558234 +1 1.76454403 +1 1.14633605 +1 -0.1210

N -1.59445102 +1 1.30129390 +1 0.50743978 +1 -0.1052

N -1.39056773 +1 -0.30302755 +1 -2.02189180 +1 -0.1168

H 1.57189674 +1 -2.30478238 +1 1.86845686 +1 0.2460

H 1.80718148 +1 -2.85647670 +1 0.44285811 +1 0.2598

C 3.37581596 +1 0.60460916 +1 0.53254622 +1 0.4356

C 2.66983340 +1 1.97800209 +1 0.57261376 +1 -0.0955

O 0.57557453 +1 2.41120948 +1 -3.25479313 +1 -0.4800

O 4.60217220 +1 0.53042449 +1 0.63891641 +1 -0.4647

O -0.04100994 +1 0.79016928 +1 4.35104465 +1 -0.4728

O -3.89448402 +1 -1.38434409 +1 1.23251158 +1 -0.4775

O -0.27145842 +1 -3.48164755 +1 -3.16931114 +1 -0.4574

H 3.26977873 +1 2.72163234 +1 1.14706661 +1 0.0621

H 2.63933509 +1 2.37988944 +1 -0.46814731 +1 0.1291

C 1.42197154 +1 1.66774038 +1 2.65170969 +1 -0.1196

H 1.32491320 +1 2.65144425 +1 3.16603749 +1 0.0701

H 2.43157032 +1 1.29349955 +1 2.94846917 +1 0.1055

C 0.40654694 +1 0.65569897 +1 3.20705661 +1 0.4093

C 0.36315048 +1 2.90399683 +1 0.78406825 +1 -0.0041

H 0.42916756 +1 3.11583351 +1 -0.31137759 +1 0.1155

H 0.65948282 +1 3.86810478 +1 1.26913785 +1 0.0407

C -1.09003575 +1 2.56945835 +1 1.19242917 +1 -0.0196

H -1.15596207 +1 2.46778954 +1 2.30316491 +1 0.0968

H -1.73896129 +1 3.45220772 +1 0.96945639 +1 0.0467

C -2.63297522 +1 0.65315587 +1 1.40792962 +1 -0.1229

H -3.61852310 +1 1.17215285 +1 1.36829582 +1 0.0669

H -2.32078022 +1 0.69838596 +1 2.48120835 +1 0.1276

C -2.79194348 +1 -0.84714032 +1 1.07539064 +1 0.4306

C -2.25745966 +1 1.70592987 +1 -0.80090000 +1 -0.0139

H -1.57912241 +1 2.38854298 +1 -1.36773833 +1 0.0968

H -3.18395193 +1 2.31140643 +1 -0.63064935 +1 0.0448

C -2.63023231 +1 0.48573983 +1 -1.67018638 +1 -0.0139

H -3.38355290 +1 -0.15647480 +1 -1.15553008 +1 0.1055

H -3.18033849 +1 0.84877071 +1 -2.57529825 +1 0.0477

C -0.69174626 +1 0.35036007 +1 -3.19681587 +1 -0.1178

H -1.39016090 +1 0.83404958 +1 -3.91758419 +1 0.0718

H -0.12849688 +1 -0.39828235 +1 -3.80548279 +1 0.1161

C 0.33846816 +1 1.35194932 +1 -2.66345522 +1 0.4162

C -1.74643630 +1 -1.71720728 +1 -2.42214331 +1 -0.1032

H -2.23102367 +1 -1.77474100 +1 -3.42361364 +1 0.0649

H -2.47286015 +1 -2.17776352 +1 -1.70932538 +1 0.1298

C -0.45284954 +1 -2.55971741 +1 -2.37250988 +1 0.4340

---------------------------------------- End of file **KOLGIB.arc**-----------------------------------

**Lutetium: RADROD**

**
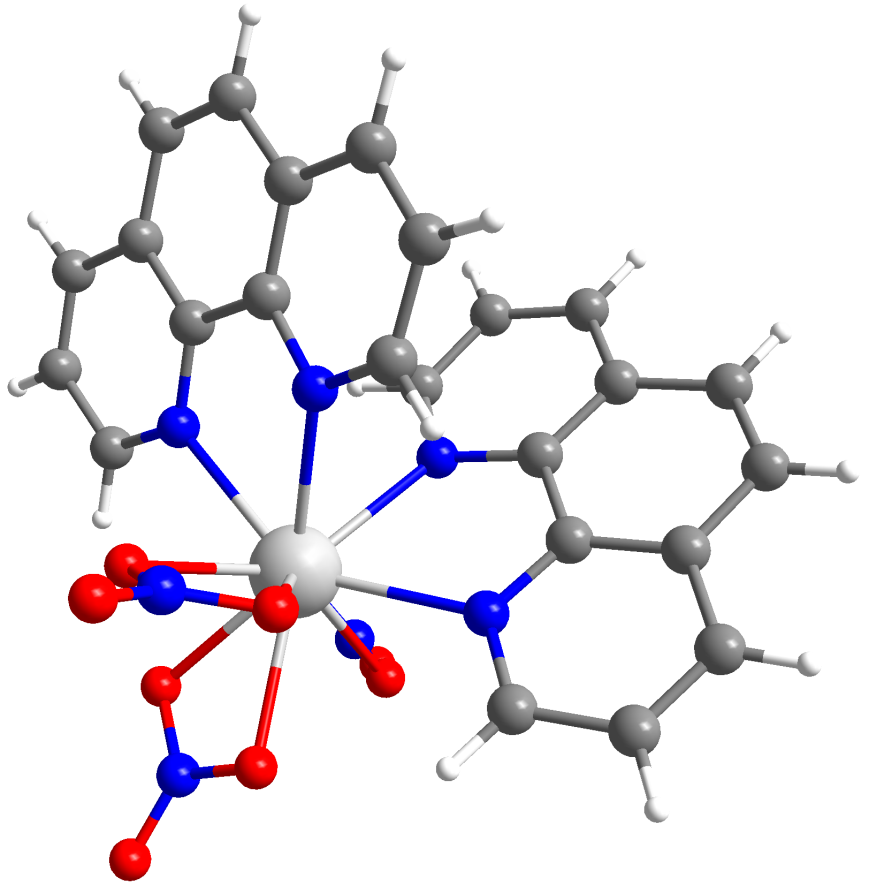
**

---------------------------------------- Begin of file **RADROD.mop**-----------------------------------

RM1 PRECISE NOINTER XYZ BFGS T=10D GNORM=0.25 +

NOLOG GEO-OK SCFCRT=1.D-10

NUMERO DE COORDENAÇÃO = 10

Lu 0.000000 1 0.000000 1 0.000000 1

N 2.477800 1 0.000000 1 0.000000 1

N 0.997714 1 2.192625 1 0.505731 1

N 0.911468 1 0.414253 1 -2.265864 1

N 0.271259 1 -2.037550 1 -1.353355 1

O 0.606928 1 0.043142 1 2.283783 1

O 0.936012 1 -1.837718 1 1.459415 1

O -1.711750 1 -1.261410 1 1.117072 1

O -1.861563 1 0.824516 1 1.274342 1

O -1.858864 1 -0.196211 1 -1.446300 1

O -1.297660 1 1.793428 1 -1.216220 1

C 3.126843 1 1.219203 1 0.000000 1

C 4.487475 1 1.376138 1 -0.251212 1

C 5.248189 1 0.258251 1 -0.572869 1

C 4.634698 1 -0.954419 1 -0.558233 1

C 3.272362 1 -1.040962 1 -0.266023 1

C 5.070986 1 2.696423 1 -0.196946 1

C 2.322962 1 2.375865 1 0.311374 1

C 2.928556 1 3.648784 1 0.411702 1

C 2.110734 1 4.735592 1 0.792344 1

C 0.808260 1 4.536310 1 1.030605 1

C 0.231456 1 3.248735 1 0.869257 1

C 4.316433 1 3.779990 1 0.140332 1

N 1.009977 1 -1.116920 1 2.471839 1

O 1.438248 1 -1.566431 1 3.516228 1

N -2.361636 1 -0.288695 1 1.579713 1

O -3.344031 1 -0.408786 1 2.236846 1

H 6.215163 1 0.339811 1 -0.797201 1

H 5.144013 1 -1.772280 1 -0.777871 1

H 2.871168 1 -1.936614 1 -0.252163 1

H 6.035439 1 2.783653 1 -0.396809 1

H 2.508877 1 5.623331 1 0.922498 1

H 0.226494 1 5.293248 1 1.293455 1

H -0.739826 1 3.120810 1 1.022610 1

H 4.696786 1 4.662873 1 0.167073 1

C 1.353347 1 -0.671561 1 -2.996817 1

C 2.110163 1 -0.569714 1 -4.160775 1

C 2.497213 1 0.688473 1 -4.606848 1

C 2.055024 1 1.772111 1 -3.916533 1

C 1.272383 1 1.596553 1 -2.773939 1

H 0.962485 1 2.405280 1 -2.312131 1

H 2.306474 1 2.682966 1 -4.205764 1

H 3.071038 1 0.795315 1 -5.414389 1

C 2.495202 1 -1.771574 1 -4.864142 1

C 2.090393 1 -2.996859 1 -4.425931 1

C 1.309951 1 -3.129699 1 -3.246970 1

C 0.966382 1 -1.973764 1 -2.511682 1

N -2.076133 1 0.986507 1 -1.757405 1

O -2.949269 1 1.381641 1 -2.504217 1

C -0.167007 1 -3.240826 1 -0.912391 1

C 0.112725 1 -4.423680 1 -1.646754 1

C 0.842721 1 -4.373499 1 -2.768022 1

H 1.019067 1 -5.192049 1 -3.281246 1

H -0.214525 1 -5.291793 1 -1.300370 1

H -0.685760 1 -3.295045 1 -0.069049 1

H 2.353925 1 -3.801053 1 -4.882124 1

H 3.047819 1 -1.673417 1 -5.677845 1

0 ---------------------------------------- End of file **RADROD.mop**-----------------------------------

---------------------------------------- Begin of file **RADROD.arc**-----------------------------------

SUMMARY OF RM1 CALCULATION, Site No: 3560

MOPAC2012 (Version: 13.357W)

Thu Feb 05 12:58:19 2015

No. of days left = 0

Empirical Formula: C24 H16 N7 O9 Lu = 57 atoms

RM1 PRECISE NOINTER XYZ BFGS T=10D GNORM=0.25 +

NOLOG GEO-OK SCFCRT=1.D-10

COORDINATION NUMBER = 10

PETERS TEST WAS SATISFIED IN BFGS OPTIMIZATION

SCF FIELD WAS ACHIEVED

HEAT OF FORMATION = -441.65675 KCAL/MOL = -1847.89184 KJ/MOL

TOTAL ENERGY = -7599.84184 EV

ELECTRONIC ENERGY = -72835.85698 EV

CORE-CORE REPULSION = 65236.01514 EV

GRADIENT NORM = 0.24659

DIPOLE = 19.11973 DEBYE POINT GROUP: C1

NO. OF FILLED LEVELS = 102

IONIZATION POTENTIAL = 10.619721 EV

HOMO LUMO ENERGIES (EV) = -10.620 -3.438

MOLECULAR WEIGHT = 721.399

COSMO AREA = 433.03 SQUARE ANGSTROMS

COSMO VOLUME = 578.51 CUBIC ANGSTROMS

MOLECULAR DIMENSIONS (Angstroms)

Atom Atom Distance

H 32 H 53 12.27921

O 49 H 28 9.77857

H 43 O 25 9.59782

SCF CALCULATIONS = 304

WALL-CLOCK TIME = 26.824 SECONDS

COMPUTATION TIME = 53.312 SECONDS

FINAL GEOMETRY OBTAINED CHARGE

RM1 PRECISE NOINTER XYZ BFGS T=10D GNORM=0.25 +

NOLOG GEO-OK SCFCRT=1.D-10

COORDINATION NUMBER = 10

Lu 0.26432217 +1 0.05032104 +1 -0.15110718 +1 -0.9052

N 2.66720491 +1 0.06704783 +1 0.13532626 +1 -0.1737

N 0.99602812 +1 2.27507534 +1 0.39791574 +1 -0.1773

N 1.22151779 +1 0.39526146 +1 -2.34486641 +1 -0.1704

N 0.52589953 +1 -2.06992760 +1 -1.25679859 +1 -0.1765

O 0.63120128 +1 0.33162394 +1 2.24996123 +1 -0.3026

O 0.95424225 +1 -1.64722361 +1 1.47156030 +1 -0.3362

O -1.56992007 +1 -1.22817942 +1 0.80588149 +1 -0.2997

O -1.67602771 +1 0.91985207 +1 1.03018798 +1 -0.3024

O -1.53191952 +1 -0.39994072 +1 -1.74639746 +1 -0.2903

O -1.06835263 +1 1.67166056 +1 -1.40356886 +1 -0.3307

C 3.21607002 +1 1.27412739 +1 0.54025577 +1 0.1060

C 4.58064024 +1 1.40715635 +1 0.82868886 +1 -0.0484

C 5.41403470 +1 0.27166959 +1 0.69137235 +1 0.0134

C 4.86482292 +1 -0.92239442 +1 0.29919166 +1 -0.1790

C 3.47446472 +1 -0.99742410 +1 0.03657211 +1 0.1455

C 5.09451797 +1 2.66430542 +1 1.27783631 +1 -0.0820

C 2.34248368 +1 2.41643376 +1 0.70561280 +1 0.1074

C 2.86914682 +1 3.61592207 +1 1.19376587 +1 -0.0568

C 1.98167020 +1 4.69642543 +1 1.42796181 +1 0.0159

C 0.64646363 +1 4.53525170 +1 1.17147911 +1 -0.1818

C 0.16909765 +1 3.30066012 +1 0.65285831 +1 0.1905

C 4.27019957 +1 3.72357670 +1 1.46159032 +1 -0.0632

N 0.85656812 +1 -0.90054216 +1 2.50620844 +1 0.5401

O 1.00056756 +1 -1.31401992 +1 3.61065951 +1 -0.1798

N -2.13923674 +1 -0.22672170 +1 1.37200488 +1 0.5397

O -3.04445526 +1 -0.35086429 +1 2.12778765 +1 -0.1588

H 6.48562721 +1 0.35329595 +1 0.90893071 +1 0.1356

H 5.47372038 +1 -1.82673910 +1 0.19648718 +1 0.1523

H 3.00555957 +1 -1.95621127 +1 -0.25004548 +1 0.1662

H 6.16795083 +1 2.74466322 +1 1.48241235 +1 0.1317

H 2.37038890 +1 5.64309655 +1 1.82136175 +1 0.1350

H -0.07592690 +1 5.33689793 +1 1.36310065 +1 0.1585

H -0.91430277 +1 3.17720689 +1 0.44644423 +1 0.2012

H 4.65846117 +1 4.68201764 +1 1.82431382 +1 0.1317

C 1.34509465 +1 -0.73978214 +1 -3.13108392 +1 0.1077

C 1.79471345 +1 -0.67572986 +1 -4.45654186 +1 -0.0488

C 2.13039949 +1 0.58887241 +1 -4.99583268 +1 0.0133

C 1.99258365 +1 1.71022336 +1 -4.21806349 +1 -0.1790

C 1.52135686 +1 1.58233503 +1 -2.88780259 +1 0.1463

H 1.37383183 +1 2.47424239 +1 -2.25218996 +1 0.1658

H 2.22625410 +1 2.70783333 +1 -4.60445019 +1 0.1522

H 2.48637566 +1 0.66231756 +1 -6.03032871 +1 0.1355

C 1.87850624 +1 -1.87058204 +1 -5.23868753 +1 -0.0820

C 1.49227817 +1 -3.06315247 +1 -4.72494149 +1 -0.0633

C 1.00572075 +1 -3.15911363 +1 -3.38313836 +1 -0.0571

C 0.95950215 +1 -2.01947795 +1 -2.57474353 +1 0.1078

N -1.91157923 +1 0.81671323 +1 -1.85093818 +1 0.5424

O -2.93142636 +1 1.13398479 +1 -2.37069313 +1 -0.1783

C 0.06472686 +1 -3.23503915 +1 -0.77699751 +1 0.1912

C 0.06772906 +1 -4.42032762 +1 -1.56206823 +1 -0.1820

C 0.54369717 +1 -4.38684347 +1 -2.84536923 +1 0.0155

H 0.56152654 +1 -5.29164819 +1 -3.46431724 +1 0.1348

H -0.32249734 +1 -5.34341621 +1 -1.11842132 +1 0.1583

H -0.32265912 +1 -3.26833924 +1 0.26247351 +1 0.2009

H 1.53842999 +1 -3.97716423 +1 -5.32792039 +1 0.1315

H 2.24958993 +1 -1.79452811 +1 -6.26682911 +1 0.1316

---------------------------------------- End of file **RADROD.arc**-----------------------------------

1. **References** ([back to contents](#Contents))
2. Allen F.H. (2002) Acta Crystallogr. B 58: 380-388.
3. Bruno I.J., Cole J.C., Edgington P.R., Kessler M., Macrae C.F., McCabe P., Pearson J., Taylor R. (2002) Acta Crystallogr. B 58: 389-397.
4. Allen F.H., Motherwell W.D.S. (2002) Acta Crystallogr. B 58: 407-422.
